# Sources, Transmission, and Control of *Salmonella* in Vertically Integrated Broiler Chicken Production: Farm, Hatchery, and Processing Plant

**DOI:** 10.1111/1541-4337.70661

**Published:** 2026-10-10

**Authors:** Deepak Subedi, Bharath Mallavarapu, Pranita Patil, Abhisek Niraula, Milan Kandel, Sanjay Kumar, Amit Kumar Singh

**Affiliations:** ^1^ School of Life and Environmental Sciences, Faculty of Science The University of Sydney Sydney New South Wales Australia; ^2^ Poultry Research Foundation The University of Sydney Camden New South Wales Australia; ^3^ Department of Poultry Science University of Georgia Athens Georgia USA; ^4^ FPL Food LLC Augusta Georgia USA; ^5^ Food Safety and Quality Assurance Department Niagara Bottling LLC Diamond Bar California USA; ^6^ Department of Agriculture and Natural Resources Delaware State University Dover Delaware USA

**Keywords:** breeder flocks, broiler production, farm to fork, food safety, genomic surveillance, hatchery, poultry processing plant, preharvest intervention, risk assessment, *Salmonella*

## Abstract

*Salmonella* remains one of the most important foodborne pathogens associated with poultry products, despite substantial progress in processing hygiene and antimicrobial interventions. This review critically examines the sources, transmission routes, and control strategies for *Salmonella* in vertically integrated broiler production systems. Attention is given to contamination originating from breeder flocks, hatcheries, feed, water, litter, grow‐out farms, transport, processing environments, and finished products. Preharvest interventions, including biosecurity, breeder and hatchery control, vaccination, litter and water management, feed hygiene, probiotics, prebiotics, organic acids, essential oils, bacteriophages, and bacteriocins, are evaluated according to mechanism, evidence strength, and commercial relevance. Postharvest interventions, including scalding, picking, evisceration control, chilling, peroxyacetic acid application, carcass rinses, post‐chill treatments, and finished‐product verification, are reviewed in relation to microbial reduction and process control. The review also discusses quantitative bio‐mapping, surveillance design, whole‐genome sequencing, predictive modeling, and quantitative microbial risk assessment as tools for identifying contamination hotspots, persistent strains, and high‐risk control points. Overall, effective *Salmonella* control requires coordinated preharvest and postharvest strategies supported by routine verification, stronger flock‐to‐product traceability, and field validation of interventions under commercial conditions.

## 1 Introduction

Broiler chicken is now the leading animal protein in the United States. US Department of Agriculture (USDA)‐ERS projects per capita broiler meat availability at 105.6 lb/person (47.9 kg) in 2026 and 106.5 lb/person (48.3 kg) in 2027, exceeding beef and pork availability, which are projected at 60.0 and 49.6 lb/person, respectively, in 2026 (USDA‐ERS 2026). This significant progress reflects decades of innovation in the poultry sector, including genetic selections for optimized growth (Decuypere et al. 2010), nutritional strategies (Ravindran and Abdollahi 2021), management, disease eradication efforts (Hitchner 2004), and vertical integration for supply chain efficiency (Young and Hobbs 2002). However, despite these advancements, poultry remains a significant contributor to foodborne salmonellosis (Thames et al. 2022).

The US Centers for Disease Control and Prevention (CDC) estimates that *Salmonella* causes approximately 1.35 million infections annually in the United States, and the latest foodborne burden estimates indicate that nontyphoidal *Salmonella* causes approximately 1.28 million domestically acquired foodborne illnesses, 12,500 hospitalizations, and 238 deaths each year (Scallan et al. 2025). Chicken continues to be a major contributor to foodborne *Salmonella* illness. The most recent Interagency Food Safety Analytics Collaboration (IFSAC) attribution report, based on outbreak data from 1998 through 2023, attributed 19.1% of foodborne *Salmonella* illnesses to chicken, making it the leading single food category in that analysis (IFSAC 2026). This persistent public health burden has continued despite longstanding regulatory and industry efforts to reduce *Salmonella* contamination in poultry products. This may reflect limitations of prevalence‐based monitoring, which does not capture pathogen concentration, serotype‐specific virulence, cross‐contamination during food handling, or low infectious doses in susceptible consumers. Although the (USDA‐FSIS 2025) framework proposed enumeration‐based process control and high‐risk serotype classification to address these gaps, the proposal was withdrawn in April 2025 for further assessment.

*Salmonella* can enter and persist in broiler production through multiple interconnected reservoirs, including breeder flocks, hatcheries, feed, water, litter, feces, poultry house environments, transport systems, processing equipment, and finished products (Shaji et al. 2023). A 2023 meta‐analysis identified hatcheries as the primary source of *Salmonella* with 48.5% prevalence, followed by litter (25.4%), feces (16.3%), and poultry house environment (7.9%). Feed (4.8%), water (2%), and the external environment of poultry houses (4.7%) further amplify risks, emphasizing the complexity of contamination pathways (Wang et al. 2023). The pooled hatchery estimate indicates that hatcheries are an important potential source of *Salmonella*, but it should not be interpreted as a precise US‐specific estimate because studies differed in location, sampling methods, and heterogeneity.

Vertical integration (Figure 1), while enhancing production efficiency, also creates vulnerabilities, as breeder farms, hatcheries, and feed mills interlink to facilitate the propagation of pathogens across the supply chain (Shaji et al. 2023). It refers to a production structure in which a single company owns or contractually controls consecutive stages of the supply chain, typically the primary breeder and pedigree stock, breeder farms, hatcheries, feed mills, contract grow‐out farms, transport fleets, slaughter and further‐processing plants, and distribution. In the US broiler chickenindustry this model accounts for essentially all commercial production. Its epidemiological significance is that it converts what would otherwise be independent operations into a connected network. A single contaminated breeder flock or feed mill can distribute *Salmonella* to hundreds of grow‐out farms through shared hatchery and feed nodes, so contamination that would be self‐limiting in a fragmented industry can propagate system‐wide. The same connectivity, however, also enables coordinated intervention and traceback that fragmented systems cannot achieve (Young and Hobbs 2002; MacDonald 2014).

**FIGURE 1:**
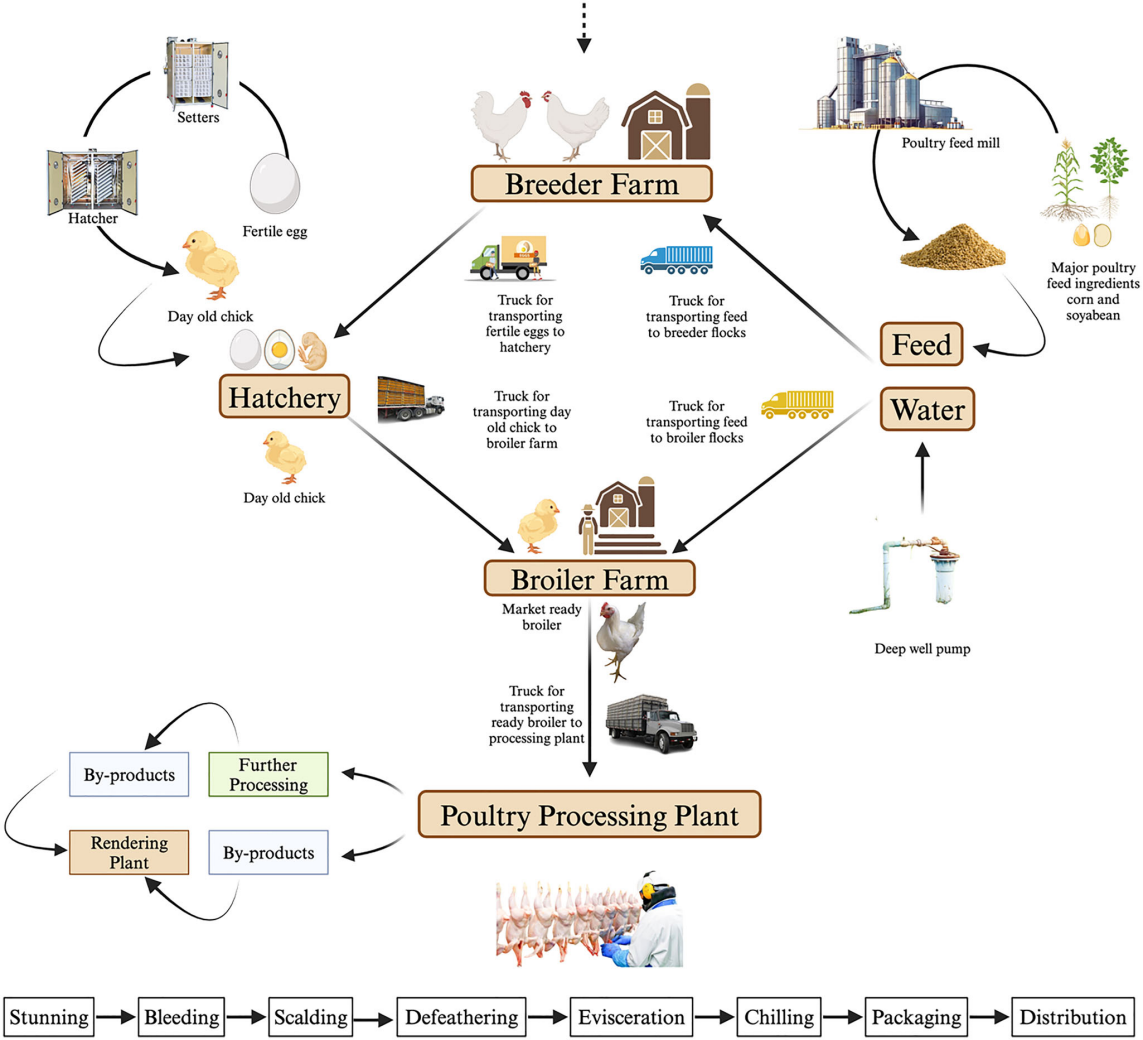
Schematic figure of a vertically integrated poultry production.

Controlling *Salmonella* demands a holistic approach. Preharvest strategies, such as biosecurity, litter management, and vaccines, aim to reduce pathogen loads in live birds, thereby minimizing cross‐contamination during processing. Postharvest antimicrobial interventions, including peroxyacetic acid and chlorine, target residual contamination. In this review, we have discussed *Salmonella* ecology across vertically integrated poultry systems and evaluated the efficacy of control measures. By aligning farm‐level practices with processing innovations, the industry can mitigate risks while meeting the continuously escalating consumer demand for safe poultry products.

## 2 Literature Search Strategy

Literature was identified through structured searches of Web of Science Core Collection, Scopus, PubMed, CAB Abstracts, and Google Scholar, supplemented by hand‐searching the reference lists of retrieved reviews and by targeted retrieval of regulatory and industry documents from US Department of Agriculture, Food Safety and Inspection Service (USDA‐FSIS), CDC, and the National Chicken Council (NCC). Searches combined terms for the organism (*Salmonella*, *Salmonella enterica*, nontyphoidal *Salmonella*), the host and production system (broiler, poultry, chicken, breeder, hatchery, grow‐out, and vertically integrated), the stage or intervention (biosecurity, litter, water treatment, feed, probiotic, prebiotic, organic acid, essential oil, bacteriophage, bacteriocin, vaccine, scalding, chilling, peroxyacetic acid, cetylpyridinium chloride (CPC), and bio‐mapping), and the outcome (prevalence, colonization, log reduction, shedding, and contamination).

The primary search covered publications from January 1990 to 2026, with earlier foundational studies included where they established a mechanism or historical event of continuing relevance, such as the 1970s *Salmonella* Agona feed‐borne outbreaks. Records were restricted to English‐language peer‐reviewed primary research, systematic reviews and meta‐analyses, and official regulatory or industry statistical publications. Studies were included if they reported quantitative or qualitative outcomes for *Salmonella* in commercial or experimental broiler systems or characterized a mechanism relevant to such systems. Studies were excluded if they addressed layer or turkey production without transferable relevance to broilers, reported only in vitro results without any in vivo or in situ correlate, concerned *Salmonella* Typhi or Paratyphi, or were conference abstracts without full peer‐reviewed publication. We emphasize that this is a comprehensive narrative review rather than a systematic review conducted to PRISMA standards.

## 3 Poultry Production in the United States

The broiler industry underwent a significant transformation during 1950s, shifting from a model dominated by small‐scale poultry farmers and independent businesses to a system marked by vertical integration. This paradigm shift consolidated operations under single entities that control multiple stages of production, processing, and marketing.

In 2022, the US poultry industry reached a milestone, producing over 9.4 billion broiler chickens (28.12 billion kg liveweight), marking a 2.9% increase from 2021. As the world's largest broiler producer, the United States exports approximately 16% of its output globally. Per capita consumption of broiler meat in 2025 was 102.9 lb (46.67 kg), whereas total chicken consumption was 104.5 lb (47.40 kg). Similarly, per capita broiler chicken consumption is projected to reach 106.2 lb (48.2 kg) in 2026 and 106.8 lb (48.4 kg) in 2027 (Figure 2; NCC 2026).

**FIGURE 2:**
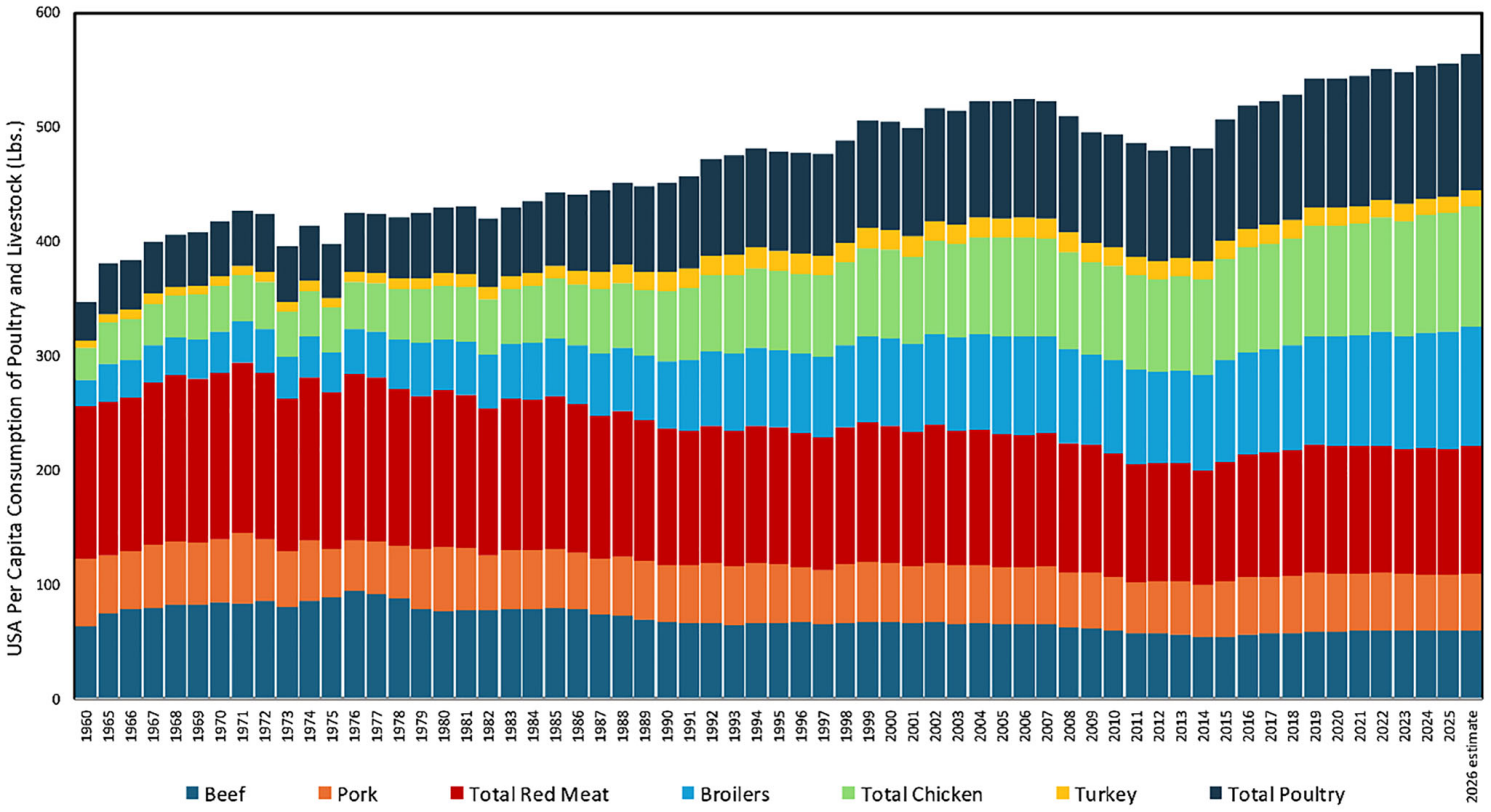
The trend chart shows the USA per capita consumption (lbs) of beef, pork, total red meat, broilers, total chicken, turkey, and total poultry from 1960 to 2026 estimate. *Source*: National Chicken Council.

Modern vertically integrated broiler enterprises manage nearly every aspect of production, including breeder flocks, hatcheries, veterinary services, feed mills, processing facilities, and transportation. This structure enables rigorous quality control, adherence to biosecurity protocols, and efficient resource management, ensuring high quality products and enhanced profitability. Currently, over 30 federally inspected companies oversee the full cycle of raising, processing, and marketing chickens, supported by approximately 20,000 family farms engaged in broiler and hatching egg production (NCC 2025).

The broiler industry comprises 160 slaughter/evisceration plants, directly employing 367,515 workers and indirectly supporting 1.4 million jobs (NCC 2025). In 2022, the total production value of broilers, eggs, turkeys, and chicken sales reached $77.0 billion, marking a notable increase of 67% from $46.1 billion in 2021 (USDA 2023a). Of this total, broilers accounted for 66%, eggs for 25%, and turkeys for 9% (USDA 2023a). The US poultry industry is the world's largest producer and the second‐largest exporter of poultry meat while also playing a significant role in egg production (NCC 2024; USDA 2023a). In 2024, broiler chicken industry exceeded 9.4 billion chickens (28.12 billion kg in liveweight), with 21.32 billion kg of chicken products being marketed, reflecting a modest 1.4% increase from 2023. The leading broiler‐producing states included North Carolina, Georgia, Arkansas, Alabama, Texas, and Mississippi. The United States exported 15% of its broiler production in 2024, with top export destinations by value being Mexico, Canada, China, Cuba, and Taiwan while by volume, the top destinations were Mexico, Cuba, Taiwan, the Philippines, and Canada (NCC 2025).

Poultry production systems are categorized into three types on the basis of the feeding programs used: conventional, No‐Antibiotics‐Ever (NAE)/Raised Without Antibiotics (RWA), and organic.

### 3.1 Conventional Production

In conventional poultry production, birds are raised on a standard feeding regimen that includes antibiotics, administered either as antibiotic growth promoters (AGPs) or ionophore anticoccidials, to enhance growth and prevent subclinical diseases at various production stages (UGA Cooperative Extension 2022).

### 3.2 NAE Production

The term “No Antibiotics Ever” (NAE) system prohibits the use of all antibiotics, including ionophore anticoccidials, throughout the bird's life (Fancher et al. 2020). NAE practices have expanded significantly, with over 50% of US broiler production adhering to these standards as of April 2019 (NCC 2021; Ritter et al. 2019). This shift is primarily driven by consumer demand for antibiotic‐free products and concerns over antibiotic resistance (Haque et al. 2020). However, producers face challenges in maintaining profitability and managing increased mortality rates. Across US broiler production, mortality rose from 3.7% in 2013 to 5.7% in 2023 (NCC 2024). Another major challenge in NAE production is disease control without compromising bird performance. Diseases, such as necrotic enteritis and coccidiosis, previously controlled by antibiotics, have re‐emerged (Fancher et al. 2020)

NAE systems are associated with increased *Salmonella* shedding and elevated mortality, both of which may reflect a greater enteric disease burden. Coccidiosis and necrotic enteritis, the two conditions most responsible for the mortality increase after ionophore and antibiotic withdrawal, both damage the intestinal mucosa. *Eimeria*‐induced epithelial damage has been shown experimentally to increase *Salmonella* invasion and cecal colonization, and concurrent coccidial infection increases the magnitude and duration of *Salmonella* fecal shedding (Qin et al. 1995). Necrotic enteritis produces comparable barrier disruption and dysbiosis (Moore 2016). Consequently, flocks experiencing the enteric challenges that drive NAE mortality would be expected to shed more *Salmonella* per surviving bird and to shed for longer than flocks in which those challenges are controlled.

Alternatives, like probiotics, prebiotics, phytobiotics, essential oils, bacteriophages, vaccines, organic acids, and enzymes, are being explored, though their efficacy varies and often falls short of antibiotics’ performance. The CDC (2019) Antibiotic Resistance Threats report highlights pathogens like Nontyphoidal *Salmonella*, *Campylobacter*, *Shigella*, and *Salmonella* Typhi as serious public health risks, emphasizing the need for One Health‐certified programs and targeted alternatives that align with NAE practices in poultry production (Ritter and ACPV 2021).

### 3.3 Organic Production

Organic poultry production prioritizes animal welfare, environmental sustainability, and disease prevention through (a) prohibiting synthetic inputs, GMOs, and routine antibiotics (USDA 2023b); (b) providing outdoor access, spacious housing, and natural behaviors; and (c) implementing robust biosecurity measures and sustainable farming practices. *Salmonella* prevalence rates in organic versus conventional systems vary across studies. Some studies found similar *Salmonella* isolation rates in both systems, with 22% (31 of 141) of conventional versus 20.8% (11 of 53) of organic chicken samples (Lestari et al. 2009). However, another study reported a higher *Salmonella* prevalence in nonorganic chickens (61.67%, 148 of 240) compared to organic chickens (37.08%, 89 of 240) on the Eastern Shore of Maryland, USA (Punchihewage‐Don et al. 2023). This difference might be attributed to the use of an updated USDA‐FSIS‐recommended whole carcass enrichment method for *Salmonella* isolation. Similarly, another study examining *Salmonella* prevalence and distribution in organic and conventional broiler poultry farms found contamination rates in fecal samples (5.6% organic vs. 38.8% conventional), feed (5.0% vs. 27.5%), and no positive water samples (Alali et al. 2010). Furthermore, higher contamination in organic processing stages (40%, 45%, 38%, and 18%) and conventional (28%, 28%, 15%, and 3%) for fecal, post‐pick, post‐evisceration, and post intra‐out body wash stages was observed, and this may be due to longer transit times of organically raised birds (Mattew et al. 2020). Similarly, a study examining retail poultry meat found that *Salmonella* was present in 5% of organic chicken samples only (Bailey and Cosby 2005), whereas another study found *Salmonella* in 27% (13 of 49) of ground chicken samples from organic farms, with none detected in conventional farms (Gad et al. 2018). These findings suggest the need for sustainable approaches to reduce *Salmonella* while balancing welfare and environmental goals (Bist et al. 2024).

Transport stress can increase *Salmonella* shedding through neuroendocrine and physiological pathways. Crating, vibration, thermal load, feed withdrawal, and social disruption activate the hypothalamic‐pituitary‐adrenal axis, elevating plasma corticosterone within minutes and sustaining it for the duration of transit (Mitchell and Kettlewell 1998). Corticosterone and the accompanying sympathetic response reduce splanchnic perfusion and increase intestinal permeability, weakening the mucosal barrier that limits *Salmonella* translocation from the gut lumen to the liver and spleen; transported birds show measurably higher rates of internal organ invasion than untransported controls (Burkholder et al. 2008). Catecholamines released during the stress response are directly exploited by *Salmonella*: noradrenaline and its bacterial‐sensed derivatives stimulate growth, motility, and the expression of virulence and invasion genes through the QseBC and QseEF two‐component systems, so the host stress response actively induced recrudescence of *Salmonella* (Bearson and Bearson 2008). Sustained corticosterone elevation is immunosuppressive, reducing heterophil function and cell‐mediated responses, which lengthens the shedding period. In addition, stress‐associated increases in intestinal motility raise fecal output within the crate, and because coops may not routinely sanitized between flocks, this simultaneously increases the external contamination load on feathers and skin.

The divergent estimates of *Salmonella* prevalence in organic and conventional broiler systems are likely attributable to methodological and production‐related confounders rather than to the rearing system alone. Important sources of variation include differences in detection sensitivity, sampling matrix, sampling stage, processing conditions, transport duration, holding time, and bird age at slaughter. For instance, whole‐carcass rinse enrichment methods may yield higher recovery rates than excised‐skin or partial‐rinse approaches, limiting direct comparison among studies. Similarly, samples collected from feces, feed, carcasses, post‐chill products, or retail meat represent different points along the production and processing chain. In addition, organic birds are often processed under different plant conditions, may experience longer transport and holding periods, and are commonly slaughtered at older ages, which may increase cumulative environmental exposure and affect *Salmonella* recovery. Therefore, comparisons between organic and conventional systems should be interpreted cautiously and adjusted for these confounding factors.

Mandatory outdoor access under the USDA National Organic Program introduces exposure pathways that are absent from fully housed systems. Wild birds, particularly passerines and waterfowl attracted to range areas and feed spillage, are recognized *Salmonella* carriers and deposit feces directly onto the range surface; wild bird isolates have been shown to share serotypes with those recovered from adjacent commercial flocks (Tizard 2004). Range soil functions as a persistent reservoir, with *Salmonella* surviving for weeks to months depending on soil type, moisture, organic amendment, and solar exposure, so that range paddocks cannot be terminally disinfected in the way that a house interior can between flocks (You et al. 2006). Standing water in range areas after rainfall provides both a survival matrix and a point of concentrated bird contact. Finally, rodents and flies move freely between the range, the house, and neighboring premises. These pathways provide the mechanistic basis for the elevated prevalence reported in some organic systems.

## 4 Vertical and Horizontal Transmission of *Salmonella* in Poultry Production

In the poultry industry, the transmission of *Salmonella* from breeder flocks to hatcheries is a crucial factor and is identified as a major contributor to *Salmonella* prevalence, with a significant 48.5% prevalence rate in broilers, highlighting hatcheries as a key intervention point (Wang et al. 2023). Transmission occurs via two primary routes: vertical and horizontal.

Horizontal transmission occurs through direct contact between chicks or indirectly via contaminated surfaces, equipment, and personnel within hatcheries (Wales and Davies 2020). Fecal–oral transmission between infected and uninfected chicks post‐hatch further facilitates spread. Inadequate biosecurity practices, including poor hygiene and ineffective disinfection, exacerbate contamination risks.

Vertical transmission occurs when colonized *Salmonella* in breeder hen is transferred to its offspring through eggs (Cui et al. 2023; Gantois et al. 2009; Figure 3). Factors, like thinner eggshells, especially in older hens, and environmental conditions (e.g., low housing temperature during oviposition), create negative pressure enabling *Salmonella* penetration (Berrang et al. 1999; De Reu et al. 2006). Serovars, such as Enteritidis, Typhimurium, Infantis, Reading, Kentucky, and others, have evolved mechanisms to persist and colonize broiler organs, including the reproductive organs (Keller et al. 1995; Raspoet et al. 2014; Wang et al. 2024). Genes, such as *csg*D and *bcs*A in *Salmonella* Enteritidis, enhance biofilm formation, increasing vertical transmission rates. Mutants lacking these genes showed reduced biofilm formation and lower rates of egg contamination (Chen et al. 2021).

**FIGURE 3:**
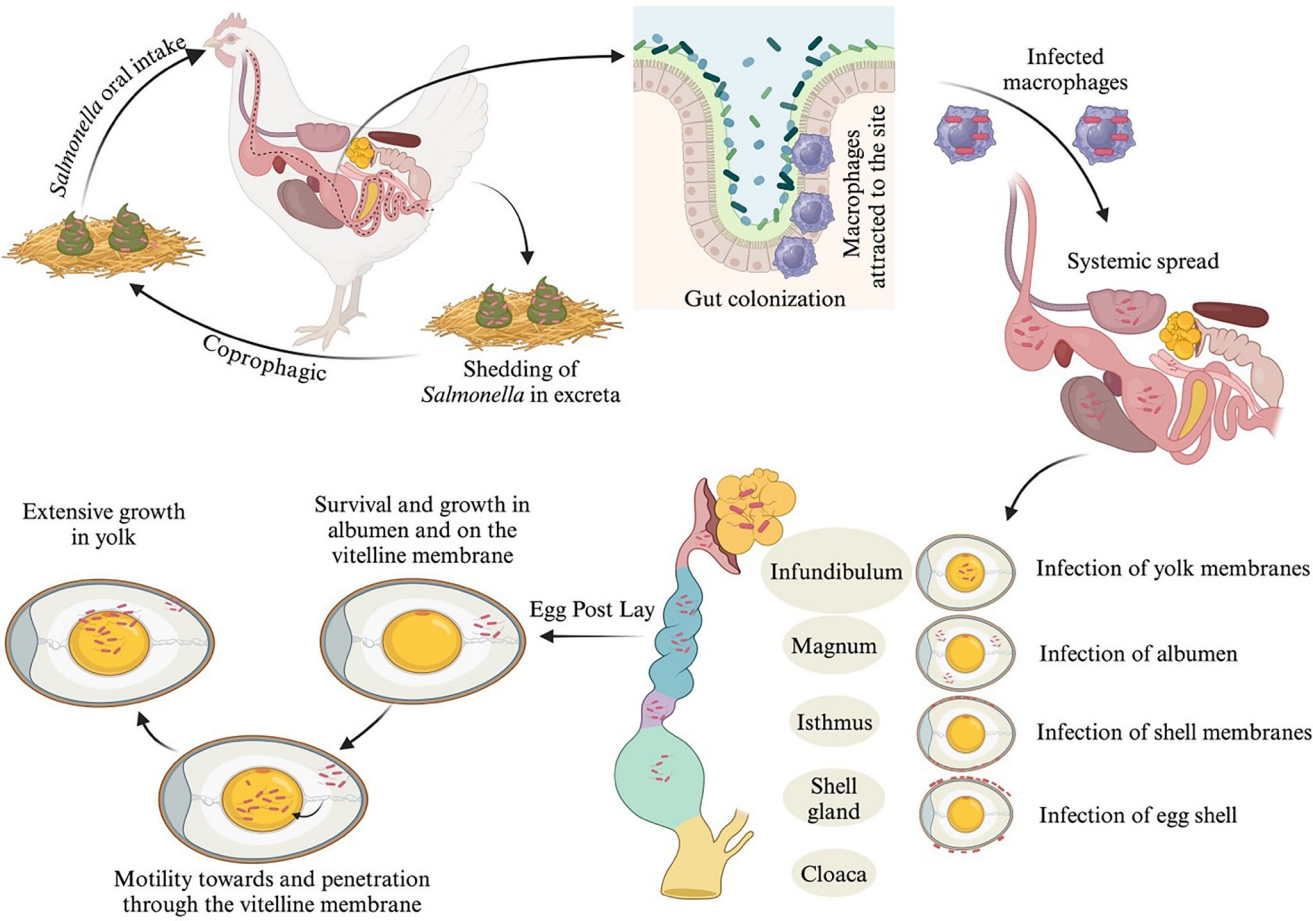
This picture illustrates the vertical transmission of *Salmonella* in poultry. The process begins with birds ingesting contaminated excreta, a behavior known as coprophagy, which leads to the shedding of *Salmonella* in feces, contributing to environmental contamination. Once ingested, the bacteria colonize the gut by interacting with epithelial cells. From the gut, *Salmonella* spreads systemically through infected macrophages, ultimately reaching the reproductive organs. In the reproductive tract, different sections are affected: the infundibulum (infection of yolk membranes), magnum (infection of albumen), isthmus (infection of shell membranes), and shell gland (contamination of the shell). Eggs can also be externally contaminated post‐lay through environmental exposure to infected feces. Once inside the egg, *Salmonella* demonstrates motility and penetrates the vitelline membrane, surviving and growing in the albumen and extensively multiplying in the yolk, which provides essential nutrients.

Notably, Kentucky, Gaminara, and Alachua are predominant serotypes in commercial hatcheries, with Enteritidis representing only 6.25% of samples (Rothrock et al. 2024). Other studies report frequent isolation of Senftenberg in hatcheries (Kim and Kim 2010; Liljebjelke et al. 2005). The absence of common serotypes across hatchery areas suggests environmental adaptation. This persistence is also influenced by *Salmonella* genetics and breeders’ genetics (Berchieri et al. 2010), underscoring the complexity of serotype identification and adaptation.

## 5 Sources of *Salmonella* in Poultry Houses

*Salmonella* colonizes the chicken gut horizontally through feed, water, reused litter, insects (darkling beetles and flies), rodents, and environmental vectors (involving other animals, wild birds, humans, vehicles, and soil) (Table 1; Figure 4; Wang et al. 2023). Even low dose (2 log CFU/g) enable gastrointestinal colonization and translocation to other internal organs (Wang et al. 2024). Once established, elimination becomes extremely challenging (Shaji et al. 2023). *Salmonella* survives in dried droppings and litter, posing risks to subsequent flocks over time (Thippareddi and Singh 2022). Therefore, a thorough understanding of the origins of the microorganism within the poultry production system and specific sources is essential to enhance control measures for *Salmonella* in poultry products (Thippareddi and Singh 2022). Poultry hatcheries are a primary source of *Salmonella* (48.5%), followed by litter (25.4%), feces (16.3%), and the internal environment of poultry houses (7.9%) (Wang et al. 2023). External environment (4.7%), feed (4.8%), chicks (4.7%), and drinking water (2%) also contribute to the prevalence of *Salmonella* (Wang et al. 2023).

**TABLE 1:** Key *Salmonella* contamination sources in poultry systems.

Source	Prevalence (%)	Key risks
Hatchery	48.5	Vertical/horizontal transmission via eggs, equipment, and aerosols
Litter	25.4	Reuse practices, beetle/rodent vectors, residual pathogens from prior flocks
Feces	16.3	Coprophagic behavior, rapid flock colonization
Poultry house environment	7.9	Dust, insects, rodents, and contaminated surfaces
Feed	4.8	Animal‐derived ingredients, poor milling hygiene
External environment	4.7	Soil, wild birds, and standing water
Water	2.0	Biofilms, additives, temperature fluctuations

**FIGURE 4:**
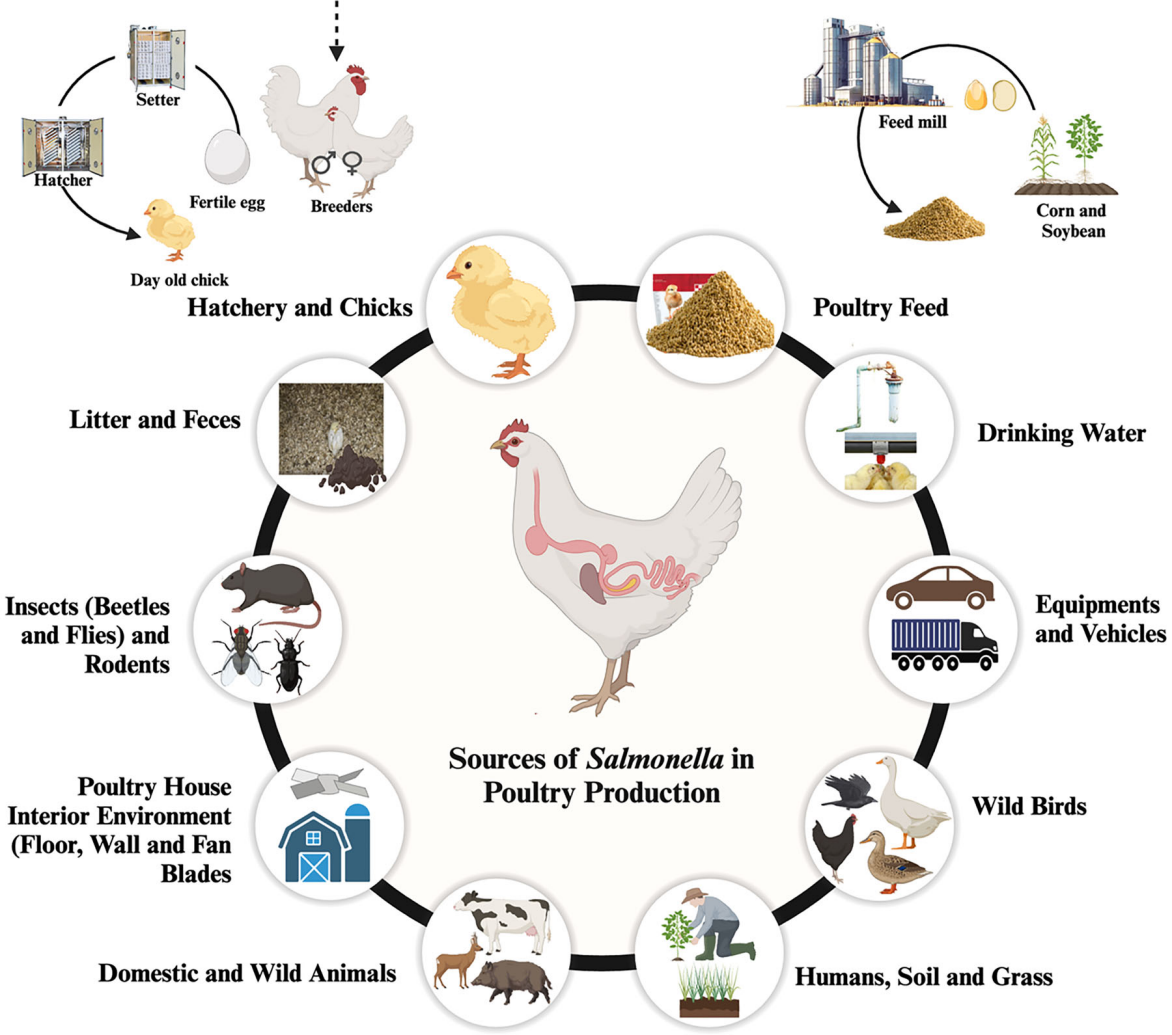
Sources of *Salmonella* in the poultry house.

### 5.1 External Environment of Broiler Houses

The external environment (soil, standing water, adjacent lands, and domestic/wild animals) serves as a *Salmonella* reservoir. *Salmonella* can survive in soil for up to 14 days (Bailey et al. 2001), and this survival is influenced by sunlight, temperature, moisture, and the presence of organic matter. Rodriguez et al. (2006) reported 12.5% *Salmonella* prevalence around poultry farms, whereas Wang et al. (2023) estimated prevalence of 4.7% in the surroundings (including puddles, wild birds, soil, and grass).

Implementing strict biosecurity measures reduces avian influenza, Newcastle disease, and *Salmonella* infections in commercial production (Delpont et al. 2023). However, even the strictest measures may not consistently controll *Salmonella* (Lu et al. 2021). Additionally, combining dietary strategies with enhanced biosecurity measures may enhance control efforts (Lu et al. 2021; Newell et al. 2011). Personnel or visitors must adhere to biosecurity measures to prevent cross‐contamination.

### 5.2 Poultry Feed

*Salmonella* infiltrates feed during harvesting, processing, and distribution, particularly for animal by‐products. *Salmonella* Agona outbreak in the USA, UK, Netherlands, and Israel in 1970 was linked to the Peruvian fishmeal (Clark et al. 1973). By 1972, it ranked 8th in the USA and 2nd in the UK, resulting in numerous human cases. The role of animal feeds like Peruvian fishmeal was highlighted in the transmission of *Salmonella* in the 1973 outbreak in Arkansas (Clark et al. 1973). *Salmonella* prevalence in raw feed components (18%), finished feed (9%), and milling equipment (8%) was reported, suggesting raw materials as the primary contamination source (Parker et al. 2022). Animal‐based raw feed components were 3.9 timesas likely to be contaminated with *Salmonella* as plant‐based components (Parker et al. 2022). Variability in *Salmonella* contamination levels within animal feed presents an ongoing challenge for the commercial feed sector. Mitigating this challenge requires proper surveillance and implementation of stringent control measures at the feed mill (Thippareddi and Singh 2022).

### 5.3 Hatchery and Breeders

Eggshell surfaces may become contaminated with *Salmonella* as eggs pass through the oviduct and cloaca (Humphrey 1994). Cooling post‐laying causes shrinkage of egg contents and potentially draws in surface pathogens. *Salmonella* can survive low‐moisture conditions, risking chick ingestion (Humphrey 1994). Eggs are contaminated with *Salmonella* through two primary routes: trans‐shell and trans‐ovarian. In trans‐ovarian contamination, eggs become contaminated before oviposition, with the source of contamination originating from the ovary and/or oviduct. Automated hatcheries inadvertently spread *Salmonella* via hatchers, dust, and fluff (Mauldin 2002; Roy et al. 2002; Chao et al. 2007).

### 5.4 Chicks

*Salmonella* transmission to chicks in a hatchery can occur both vertically and horizontally. Although vertical transmission is possible, the primary route likely involves eggshell contamination and aerosolization during hatching (Shaji et al. 2023; Liljebjelke et al. 2005). Chicks ingest contaminated membranes or respiratory exposure to aerosols and fluff (Mitchell et al. 2002). Immature microbiota and immunity make day‐old chicks susceptible to *Salmonella* colonization (Cox et al. 1990). Wang et al. (2023) estimated 48.5% *Salmonella* prevalence in hatcheries (e.g., equipment surfaces, eggs, tray liners, eggshells, and fluff) and 4.7% in chicks.

### 5.5 Water

Poultry drinking water supply on poultry farms sourced from municipal supply or wells carries minimal risk despite reported instances of contamination with foodborne pathogens (Maharjan, Dey, et al. 2017). However, this minimal risk can be compromised due to warm temperature (27–30°C), low flow, and the inclusion of water additives such as medications (Raut et al. 2024). The prevalence of *Salmonella* in poultry water ranged from 0% to 11% across all regions (Wang et al. 2023). Drinking water treatment with chlorine dioxide or including organic acids (Parker et al. 2006; Wang et al. 2025) limits the horizontal transmission of *Salmonella*.

### 5.6 Bird Dropping (Excreta) and Litter

Chicks can be exposed to *Salmonella* shortly after hatching via respiratory, oral, or intra‐cloacal routes (Cox et al. 1996). Once colonized, chicks excrete high levels of *Salmonella* in the litter during grow out, facilitating the spread of the pathogen within the flock via ingestion of excreta and litter. Thus, litter sampling using drag swabs or boot swabs is a reliable method for determining bird and flock colonization status, though not all birds within the flock become colonized (Buhr et al. 2007; Kyakuwaire et al. 2019). Reusing litter in poultry houses is a common practice in the United States, driven by economic considerations and evidence suggesting potential benefits for broilers, including increased body weight, daily weight gain, feed consumption, and production efficiency (Merino et al. 2018). However, reused litter poses a risk of *Salmonella* cross‐contamination from sources such as darkling beetles, rodents, or residual pathogens from previous flocks. Colonized birds shed *Salmonella* into the litter, increasing the risk of transmission to other birds in the flock. Furthermore, *Salmonella* can persist in dry litter between flock cycles, potentially infecting subsequent batches of chicks.

### 5.7 Poultry House Interior Environment

Poultry house interiors act as significant reservoirs for *Salmonella* and *Campylobacter* transmission, facilitated by vectors like darkling beetles, flies, and rodents (Hazeleger et al. 2008; Wales et al. 2010). Colonization of these pathogens in bird gut leads to fecal shedding, perpetuating contamination cycles within flocks (Gast and Holt 1998). *Salmonella* and *Campylobacter* can be transmitted via beetles and their larvae across successive rearing cycles, with *Salmonella* persisting in beetles for up to 2 weeks between broiler flocks (Skov et al. 2004; Hazeleger et al. 2008). Rodents, particularly mice, pose persistent challenges in poultry environments, as their guts serve as *Salmonella* reservoirs (Meerburg and Kijlstra 2007). Contaminated rodent droppings contribute to transmission, with prevalence rates of up to 40% reported in poultry house roof rats (Bailey et al. 2001). Additionally, dust on fan blades and walls, harbor *Salmonella*, posing contamination risk to birds and vectors (Bailey et al. 2001, 2002).

Exposure to *Salmonella* can occur through multiple sources, including litter, drinkers, and chickens’ natural coprophagic behavior (Shaji et al. 2023). Once colonization occurs, even in a small subset of birds, colonization may spread within the flock,  increasing prevalence across the flocks. Although *Salmonella* concentrations in excreta may decline over time, the bacteria can persist in bird gut, heightening cross‐contamination risk during processing (Maciorowski et al. 2004). Reducing pathogen populations in the gut and on surfaces prior to slaughter is critical to mitigating this risk. Addressing *Salmonella* contamination at the preharvest stage (e.g., farm level interventions) is imperative, complementing existing efforts to minimize risks during broiler processing.

## 6 Control Strategies to Reduce *Salmonella* in Broiler Flocks

### 6.1 Biosecurity and Poultry House Management

Although eliminating *Salmonella* sources from the US poultry grow‐out environment remains challenging, effective measures can minimize pathogen introduction into the poultry house interiors and subsequent bird exposure (Thippareddi and Singh 2022). Comprehensive management strategies include maintaining *Salmonella*‐free breeding flock, rigorous hatchery protocol, facility cleaning, pest control, strict biosecurity, and decontamination of litter, feed, and water (Obe et al. 2023; Figure 5).

**FIGURE 5:**
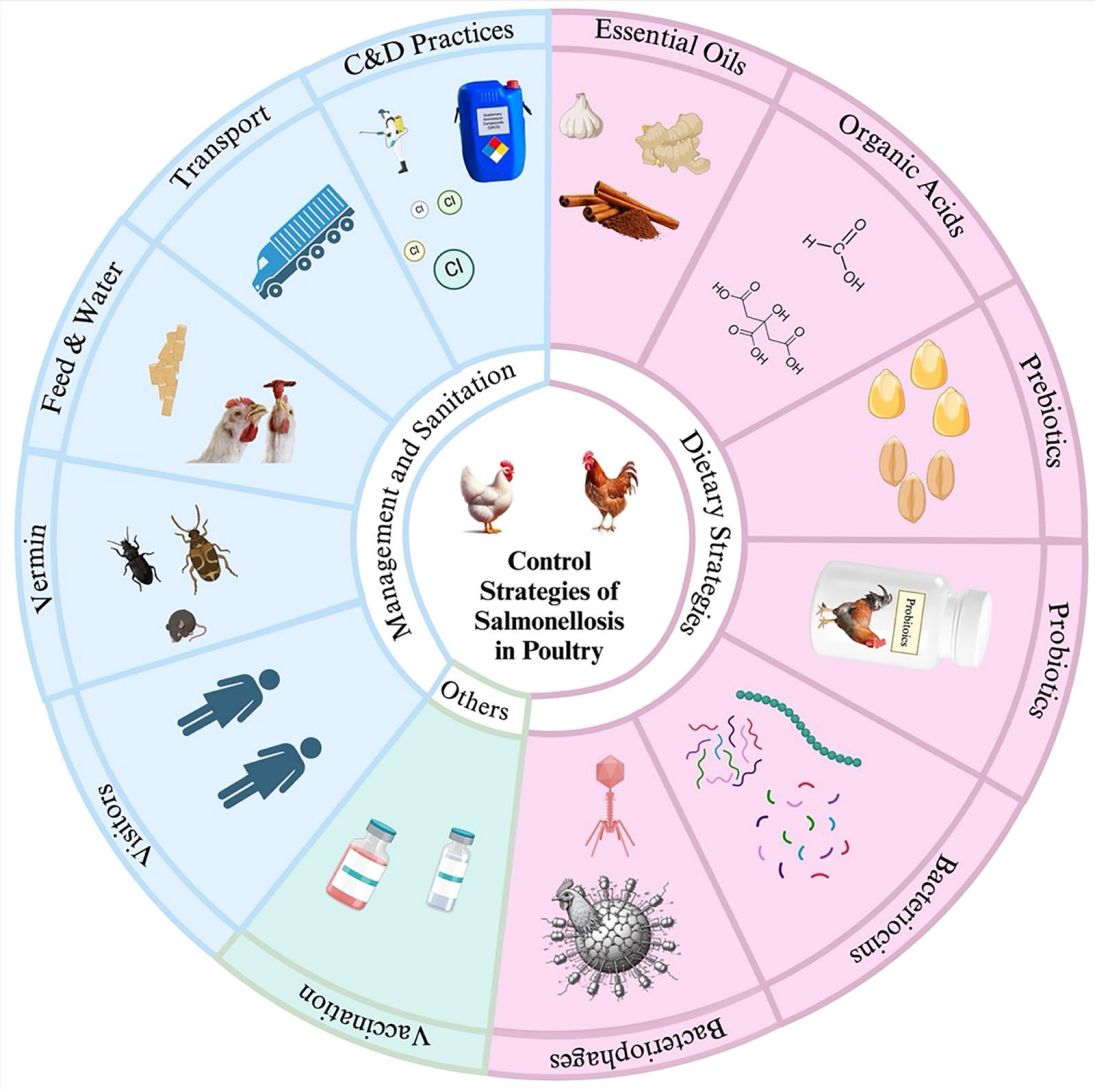
Control strategies across breeder and broiler production systems to minimize the risk of *Salmonella* contamination in broiler chicken meat.

#### 6.1.1 Biosecurity

Biosecurity is critical in poultry production for *Salmonella* control, as horizontal transmission within flocks becomes difficult to manage once the pathogen colonizes the bird gut ecosystem. A robust biosecurity plan, including stringent personnel hygiene protocols (e.g., thorough footwear cleaning and sanitizing, providing boot dips) before entering poultry houses. Additionally, all equipment entering the facility must undergo proper sanitation to prevent pathogen spread.

Biosecurity encompasses practices that prevent or reduce the introduction and transmission of pathogens, including *Salmonella*, on farms (Conan et al. 2012). The key measures include (a) cleaning and disinfecting farm equipment, used for operations both inside and outside the poultry house, footwear, protective clothing, and vehicles visiting the farm and (b) implementing a four‐step cleaning and disinfection program in grow‐out houses: (1) dry and wet cleaning to remove debris and organic matter, (2) proper use of disinfectants to eliminate microorganisms, (3) rinsing to remove residues, and (4) fumigation. Common disinfectants include aldehydes, peroxides, quaternary ammonium compounds, and phenolic substances, with their effectiveness depending on the procedures and correct application (Obe et al. 2023). Additionally, pest control is equally vital. Rodents and insects can serve as mechanical and biological vectors for introducing and spreading *Salmonella* within poultry houses and across farms (Meerburg and Kijlstra 2007). Effective strategies include physical barriers to prevent pest access, use of traps, bait stations, and vegetation removal around the house, and rotational use of insecticides and rodenticides (Van Immerseel et al. 2009). *Salmonella* can spread via vertical transmission, leading to bacterial contamination of eggs and potentially *Salmonella*‐positive chicks. To mitigate this, sourcing eggs from *Salmonella*‐free breeder flocks is ideal (Gast 2007). Surface contamination from feces or air is also a risk. Egg sanitization methods that strip the egg cuticle are discouraged due to increased vulnerability to penetration and reduced hatchability, whereas alternatives like ultraviolet irradiation should be used, which can effectively reduce contamination without compromising hatchability (Coufal et al. 2003).

#### 6.1.2 Litter Management

Reusing poultry litter is a common practice in the US poultry industry, driven by economic benefits and environmental sustainability goals. Effective litter management is critical, as birds remain in continuous contact with litter throughout their growth cycle. Litter quality directly impacts poultry health, productivity, and welfare, with poor quality linked to microbial proliferation and conditions such as pododermatitis, breast abrasions, and keratoconjunctivitis, all of which compromisecarcass quality (Namata et al. 2008; Volkova et al. 2010; Bodí et al. 2013). Deterioration of litter, often caused by mishandling, inadequate ventilation, or excessive moisture, creates favorable conditions for pathogenic microorganisms like *Salmonella* to thrive (Bodí et al. 2013). Critically, litter can serve as a reservoir for *Salmonella* from prior flocks, highlighting the importance of proper management practices to minimize the cross‐contamination risk (Talorico 2021).

Water activity governs *Salmonella* behavior in litter, but the threshold for growth and the threshold for survival differ substantially and should not be conflated. The minimum water activity permitting growth of *Salmonella* is approximately 0.94 under otherwise optimal conditions, and practical control targets are set well below it. Field data map these values onto flock outcomes: in broiler houses, dry litter surfaces with water activity of 0.90–0.95 were associated with *Salmonella*‐positive flocks by drag swab, values of 0.85–0.89 defined a transitional band of increased risk, and values of 0.79–0.84 were associated with *Salmonella*‐negative flocks (Opara et al. 1992). Maintaining litter water activity below approximately 0.84, corresponding broadly to a moisture level of 20%–30%, therefore represents a defensible operational target for suppressing foodborne pathogen growth. Crucially, however, low water activity suppresses multiplication without achieving inactivation: *Salmonella* enters a desiccation‐tolerant state in dry litter and organic matter, upregulating osmotic and oxidative stress responses, and can persist for weeks to months at values far below those permitting growth (Finn et al. 2013; Gutierrez and Schneider 2022). Dry litter management is thus a growth‐limiting measure, not a decontamination step, and must be paired with composting, windrowing, or downtime if reduction rather than containment is the objective (Eriksson De Rezende et al. 2001; Watkins 2008). The composting process involves creating windrows of litter and periodically turning them to sustain temperatures exceeding 130°F (54°C), which effectively reduces *Salmonella* populations (Lavergne et al. 2006). Composting alters microbial communities, suppressing pathogens while significantly reducing aerobic and anaerobic bacteria by 1–2 log CFU (Macklin et al. 2006, 2008). In‐house composting using non‐breathable traps and achieving temperatures above 122°F (50°C) for at least 24 h helps eliminate *Salmonella* in treated litter (Macklin et al. 2008).

Reported time–temperature targets should distinguish laboratory thermal inactivation from in‐house windrowing. Although the US Environmental Protection Agency (USEPA) Process to Further Reduce Pathogens standard requires windrow composting at ≥55°C for 15 days with at least five turnings, this standard should not be directly equated with short‐cycle in‐house poultry litter windrowing (USEPA 2003). In‐house studies show that windrowing can raise core litter temperatures to approximately 50–55°C within 24–48 h and reduce bacterial counts or pathogen recovery; however, a consistently validated 5‐log reduction of *Salmonella* has not been demonstrated under commercial in‐house conditions (Macklin et al. 2006; Brooks et al. 2015). Because heating is spatially uneven, turning is important to move cooler surface and edge material into the thermal core.

Downtime between flocks may further reduce *Salmonella* through desiccation, ammonia accumulation, temperature exposure, and microbial competition; however, ambient downtime alone should not be considered an elimination step. *Salmonella* populations in litter decline approximately log‐linearly during downtime, with the steepest reduction in the first 7–10 days, after which the surviving desiccation‐adapted subpopulation declines slowly and may persist for more than 100 days (Payne et al. 2007; Roll et al. 2011). A 10‐ to 14‐days downtime period may be a practical operational target, extended after high‐risk flocks where feasible, but the optimal duration remains insufficiently defined. Therefore, downtime should be combined with litter treatment or windrowing rather than used as a stand‐alone control.

#### 6.1.3 Water Treatment

Water is critical for poultry metabolism, waste excretion, and overall productivity (Bell 2002; Jafari et al. 2006). Birds consume approximately twice as much water as feed, with intake influenced by age, species, activity level, environmental conditions (e.g., temperature and humidity), respiratory rate, and health (Mottet and Tempio 2017; Orakpoghenor et al. 2021). Deviations from normal water consumption patterns may indicate health issues (Raut et al. 2024). Ensuring birds have adequate access to clean water is essential for their health and productivity. Microbial contamination in poultry water systems, exacerbated by warm temperatures and low‐flow rates, poses health risks (Maharjan 2013). Bacterial biofilms in waterlines resist disinfection, increasing pathogen persistence, present challenges, and can harm bird health (Maharjan, Huff, et al. 2017). Poor water quality can lead to leaky water nipples, wetting the litter, increasing ammonia levels, and ultimately reducing bird performance and livability. This highlights the importance of treating poultry drinking water to maintain flock health and optimize performance (Fairchild and Ritz 2015). Effective sanitation of poultry drinking systems is essential for preharvest food safety as it can minimize the risk of foodborne pathogens and protect consumer health while enhancing flock performance. Sodium hypochlorite (NaOCl), chlorine gas (Cl_2_), and calcium hypochlorite (Ca(ClO)_2_) are commonly used disinfectants for drinking water sanitation in poultry operations. These substances generate hypochlorous acid (HOCl) through hydrolysis, which has a strong germicidal action and is effective at pH 6.5–7.5 (USEPA 1999).

Alternatively, hydrogen peroxide (H_2_O_2_; 25–50 ppm residual) and chlorine dioxide (ClO_2_), ozone, and heavy metal ions (e.g., silver and copper) disrupt microbial cell walls/metabolism (Cho et al. 2010; Park et al. 2009). Free chlorine (2–5 ppm) in drinking water is also effective in reducing microbial counts (Maharjan, Dey, et al. 2017).

Hydrogen peroxide is markedly less stable in poultry waterlines than its in vitro efficacy suggests. Catalase and peroxidase activity from organic matter, feed particles, and the established bacterial population decomposes hydrogen peroxide to water and oxygen, so measured residuals fall rapidly with distance from the injection point; in heavily fouled lines, residual can approach zero at the far end of the house, whereas the proximal end remains at target concentration. Hydrogen peroxide also reacts nonspecifically with the extracellular polymeric matrix of established biofilms, so the applied dose is consumed at the biofilm surface before reaching the embedded cells, and biofilm‐associated *Salmonella* can be one to three orders of magnitude less susceptible than planktonic cells (Bridier et al. 2011).

Physical methods include ultraviolet rays and ultrasonic waves, which inactivate pathogens without chemical residues (Hijnen et al. 2006; Phull et al. 1997). These should not be assumed universally applicable, because both are strongly constrained by water quality. Ultraviolet disinfection depends on delivered fluence at 254 nm, and transmittance declines exponentially with turbidity, dissolved organic carbon, and dissolved iron and manganese, all of which are common in well water used on poultry farms. Suspended particles additionally shield microorganisms physically: Cells associated with or embedded within particles larger than approximately 10 µm can survive fluences that inactivate free‐swimming cells by several logs, so measured log reduction in turbid water can fall well below the design value even when lamp output is nominal (Kollu and Ormeci 2012). Iron deposition and biofilm growth on quartz sleeves further reduce delivered fluence over time unless sleeves are cleaned on a defined schedule. Ultrasonic treatment is similarly limited: Cavitation intensity attenuates with distance from the transducer and is dissipated by suspended solids, and at the power densities and flow rates practical for farm‐scale waterlines, it typically achieves less than 1 log reduction as a stand‐alone treatment (Joyce et al. 2003). Both technologies therefore require upstream filtration and in practice function as adjuncts that reduce the chemical demand of a primary disinfectant rather than as replacements for it. Neither provides a persistent residual, so neither controls regrowth or biofilm formation downstream of the treatment point, which is a decisive limitation in the long, low‐flow waterlines characteristic of broiler houses.

Enhanced effectiveness is achieved by initially eliminating organic matter before applying the disinfectant. Contaminated water increases the prevalence of foodborne pathogens like *Salmonella*, *Campylobacter*, and *Listeria monocytogenes*, some of which exhibit resistance to disinfectants (Cook et al. 2012). Effective water treatment mitigates risks, safeguarding both flock health and consumer safety.

#### 6.1.4 Poultry Feed Hygiene

Poultry feed is a significant vector for pathogens such as *Salmonella*. Efforts to mitigate *Salmonella* contamination span pre‐ and postharvest stages, emphasizing preharvest interventions at feed mills. Preventive measures start by sourcing raw ingredients from certified suppliers (Muckey 2016), inspecting incoming loads, and enforcing strict segregation between “clean” (processed) and “dirty” (raw) zones minimizes cross‐contamination (Morita et al. 2006; Obe et al. 2023). The risk of pathogen transmission can also be reduced during operations by using antibacterial foot baths or personal protective equipment (PPE) during transitions between zones. Similarly, control of dust and regular cleaning are crucial, as dust is a major source of contamination, and spilled ingredients can attract pests (Jones 2011).

Thermal processing during pelleting effectively reduces pathogen load; however, it may risk nutrient degradation and post‐process contamination during cooling, packaging, and transportation to farms. The application of chemical feed additives (formaldehyde, organic acids, and essential oils) can further control pathogens but raise concerns related to costs, labeling, and worker safety (Cochrane et al. 2016; Muckey 2016). A holistic approach, including ingredient monitoring, equipment maintenance, thermal processing, and chemical treatments, is critical to minimizing *Salmonella* contamination in feed, ultimately reducing the spread of *Salmonella* to poultry flocks and protecting consumers.

### 6.2 Dietary Strategies

For the past 50 years, subtherapeutic antibiotic supplementation in poultry feed has been a common practice to enhance gut health and suppress both pathogenic and nonpathogenic enteric microorganisms (Abreu et al. 2023). However, shifts in consumer awareness and regulatory pressure over antibiotic‐resistant gene transfer in foodborne pathogens have driven the industry to adopt antibiotic‐free (ABF) production systems (Abreu et al. 2023). About 46% of US poultry meat is now produced under the ABF system (Thippareddi and Singh 2022), prompting the search for alternative feed additives to maintain gut health and control pathogens like *Salmonella* and *Campylobacter*. Various strategies have been assessed are organic acids, probiotics, prebiotics, botanicals, bacteriocins, bacteriophages, and combinations of feed additives to improve the gut health of the bird and control foodborne illnesses such as *Salmonella* (Shaji et al. 2023; Singh et al. 2025). A single approach cannot completely eliminate the *Salmonella* risk in poultry, necessitating integrated control programs that combine approaches.

#### 6.2.1 Probiotics

Probiotics or direct fed microbials (DFM) are live microorganisms that can confer various benefits to the host, mainly through competitive exclusion of pathogens and modulation of host immune systems (Jha et al. 2020). Probiotics may reduce *Salmonella* colonization by competing for epithelial adhesion sites through surface adhesins, mucus‐binding proteins, and S‐layer proteins, thereby limiting attachment and invasion (Van Tassell and Miller 2011). They may also compete for nutrients used by *Salmonella* in the inflamed gut, including tetrathionate, fucose, and sialic acid, reducing its metabolic advantage (Winter et al. 2010; Ng et al. 2013). Some lactic acid bacteria produce bacteriocins or other antimicrobial compounds with activity against *Salmonella*, whereas fermentation‐derived lactate and short‐chain fatty acids (SCFAs) lower luminal pH and increase intracellular acid stress; butyrate can also repress SPI‐1 expression and reduce invasion capacity (Gantois et al. 2006). In addition, probiotics may modulate host immunity by enhancing secretory IgA, host defense peptides, and tight junction integrity. Lactic acid bacteria have been extensively characterized for their capacity to augment the animal immune system, eliciting beneficial effects on both innate and adaptive immune responses (Ashraf and Shah 2014; Tellez et al. 2012).

Probiotics can be administered to birds through feed or water, either as single strains or as a mixture. The most common probiotics used are lactic acid bacteria, which include the genera *Lactobacillus* (Shi et al. 2022), *Lactococcus*, and *Bifidobacterium*. Other microorganisms, such as *Bacillus* (Biswas et al. 2023; Girgis et al. 2022), *Enterococcus* (Shao et al. 2022), molds (Azeem et al. 2019), and yeasts (Pang et al. 2022), have also been studied for their potential health benefits as feed supplements in poultry. Supplementation of feed with *Bacillus subtilis* B2A significantly improved feed intake and feed conversion, increased the bursa of Fabricius weight, reduced *Salmonella* population in the intestine, and enhanced breast meat quality by increasing water‐holding capacity and reducing drip loss (Park and Kim 2014). Similarly, administration of *Lactobacillus reuteri* S5 from an early age improved microbial species diversity in the chicken ceca, increased the abundance of beneficial bacteria, reduced *Salmonella* population, strengthened the intestinal barrier, and enhanced overall gut health (Shi et al. 2022). A combination of *Bacillus subtilis*, *Clostridium butyricum*, and *Enterococcus faecalis* significantly improved growth, immune organ development, and carcass traits of male broilers and also reduced fecal *Salmonella* up to 2.61 log CFU/g (Zou, Meng, et al. 2022). Similarly, a blend of *Bacillus* spp. and *Saccharomyces cerevisiae* improved intestinal morphology and immune responses, lowering cecal *Salmonella* by 0.79 log CFU/g (Fazelnia et al. 2021; Table 2). More recently, Fenster et al. (2026) reported that a direct‐fed microbial and xylanase enzyme blend reduced *Salmonella* incidence by approximately 30% in broiler chickens, while also improving antioxidant responses and performance parameters, highlighting the potential of microbial‐based feed additives to improve both gut health and food safety.

**TABLE 2:** Probiotic‐based strategies evaluated for controlling *Salmonella* colonization in broiler chickens, with or without *Salmonella* challenge.

Probiotic	Probiotic dose	*Salmonella* serotype	Challenge day and dose	Rearing condition and broiler type	Major findings	References
Mix probiotics (*Bacillus subtilis*, *Clostridium butyricum*, and *Enterococcus faecalis*)	0.5 g/kg (0.05%) of *B. subtilis* (2 × 10^8 CFU/g), *C. butyricum* (2 × 10^6 CFU/g), and *E. faecalis* (1 × 10^6 CFU/g) in feed	No challenge	N/A	Pens; AA+ broilers	The probiotic complex significantly improved growth and carcass performance, reduced NH_3_ and H_2_S emissions, and decreased fecal *Salmonella* counts by 1.97 log CFU/g	Zou, Fan, et al. (2022)
Mix probiotics (*B. subtilis*, *C. butyricum*, and *E. faecalis*)	2 g/kg (0.2%) of *B. subtilis* (2 × 10^8 CFU/g), *C. butyricum* (2 × 10^6 CFU/g), and *E. faecalis* (1 × 10^6 CFU/g) in feed	No challenge	N/A feces	Pens; AA+ broilers	Probiotic complex enhanced immune organ development, especially spleen, thymus, and bursa, and significantly reduced *Salmonella* population by 2.61 log CFU/g in the feces	Zou, Meng, et al. (2022)
Mix probiotics (*Bacillus* spp. and *Saccharomyces cerevisiae*)	0.2 g/kg feed of probiotic blend containing *B. subtilis* 4 × 10^8 CFU/g, *Bacillus licheniformis* 3 × 10^9 CFU/g, and *S. cerevisiae* 1 × 10^8 CFU/g	Typhimurium	21 days; 0.5 mL 1 × 10^6 CFU/mL/bird orally	Cages; Ross 308 broilers	The probiotic complex enhanced growth performance and immune responses in chicks, improved intestinal morphology by competing with *Salmonella* adhesion sites, and reduced cecal *Salmonella* counts by 0.79 log CFU/g	Fazelnia et al. (2021)
*Bacillus subtilis* mix 3 strains (NP122, B2, and AM0904)	0.5 g/kg feed of probiotic blend containing *B. subtilis* 3 strains (NP122, B2, and AM0904) supplying 2.0 × 10^6 CFU/kg feed	Heidelberg	3 days; 1 mL of 1 × 10^7 CFU/g/bird orally	Cages; Cobb 500 male broilers	The probiotic complex promoted a more diverse cecal microbiota, contributing to improved gut health and growth performance, and significantly reduced cecal *Salmonella* counts by 1.13 log CFU/g	Hayashi et al. (2018)
Mix probiotics (*Lactobacillus acidophilus*, *B. subtilis*, and *Aspergillus niger*)	340 g/kg (34%) fermented rapeseed meal (FRSM) in feed with 10^5 CFU mixed culture of *L. acidophilus* (PTCC1643), *B. subtilis* (PTCC1156), and *A. niger* (PTCC5010) per kg FRSM	Typhimurium	3 days; 0.5 mL of 1 × 10^5 CFU/bird orally	Pens; Cobb 500 male broilers	The probiotic mix in FRSM improved weight gain and feed conversion ratio, resulted in the lowest heterophil counts and H/L ratios, and significantly reduced cecal *Salmonella* counts by 0.654 log CFU/g	Ashayerizadeh et al. (2017)
Mix lactic acid bacteria (LAB) probiotics	0.2 g/kg (0.02%) feed of LAB containing 5.2 × 10^10 CFU/g of *L. acidophilus*, *Lactobacillus casei*, *Bifidobacterium thermophilum*, and *Enterococcus faecium*	Typhimurium	3 days; 0.5 mL 1 × 10^6 CFU/mL/bird orally	Cages; Ross 308 broilers	The LAB mix reduced *Salmonella* colonization in the gut and internal organs, enhanced intestinal mucosal morphology, and significantly decreased cecal *Salmonella* counts by 0.3 log CFU/g	Jazi et al. (2019)
*B. subtilis* and *B. licheniformis*	*B. subtilis* @ 1.4 × 10^9 CFU/kg feed, and *B. licheniformis* 8.2 × 10^8 CFU/kg feed	No challenge	N/A	Pens; Ross 308 male broilers	The probiotic mix of *B. subtilis* and *B. licheniformis* improved growth performance, reduced feed conversion ratio without affecting feed intake or mortality, decreased NH_3_, H_2_S, and acetic acid emissions, increased *Lactobacillus*, and reduced cecal *Salmonella* counts by 0.08 log CFU/g	Biswas et al. (2023)
*B. subtilis* B2A	*B. subtilis* B2A @ 1 × 10^5 CFU/g of feed	No challenge	N/A	Cages; Ross 308 male broilers	Dietary supplementation with *B. subtilis* did not affect white blood cell, lymphocyte, or haptoglobin levels, but significantly reduced *Salmonella* counts in the large intestine by 1.24 log CFU/g	Park et al. (2014)
*B. subtilis* RX7	1 g/kg (0.1%) feed of probiotic blend containing *B. subtilis* RX7 1.0 × 10^9 CFU/g	No challenge	N/A	Cages; Ross 308 broilers	Supplementation with *B. subtilis* RX7 significantly increased weight gain, reduced serum haptoglobin concentrations, and decreased cecal *Salmonella* counts by 0.49 log CFU/g	Park et al. (2018)
*B. subtilis* C14	1 g/kg (0.1%) feed of probiotic blend containing *B. subtilis* C14 1.0 × 10^9 CFU/g	No challenge	N/A	Cages; Ross 308 broilers	Supplementation with *B. subtilis* C14 significantly increased weight gain, reduced serum haptoglobin levels, and decreased cecal *Salmonella* counts by 0.42 log CFU/g	Park et al. (2018)
*B. subtilis*	0.25 g/kg (0.025%) of *B. subtilis* preparation containing 2.0 × 10^10 CFU/g in feed	No challenge	N/A	Cages; Arbor Acres (AA) broilers	Supplementation of *B. subtilis* improved growth performance and reduced the *Salmonella* population by 0.35 log CFU/g	Gao et al. (2017)
*B. subtilis*	0.2 g/kg (0.02%) of *B. subtilis* preparation containing 2.0 × 10^10 CFU/g in feed	No challenge	N/A	Cages; Arbor Acres (AA) broilers	Supplementation of *B. subtilis* improved growth performance and significantly reduced the *Salmonella* population by 0.88 log CFU/g	Gao et al. (2017)
*B. subtilis*	1 g/kg (0.1%) of *B. subtilis* containing 6 × 10^10 CFU/g in feed	No challenge	N/A	Cages; Arbor Acres broilers	Supplementation of *B. subtilis* improved growth performance and significantly reduced the *Salmonella* population by 0.75 log CFU/g	Li et al. (2008)
*Lactobacillus reuteri*	1 g/kg (0.1%) commercial DFM containing *L. reuteri* in feed	No challenge	N/A	Pens; Ross 308 broilers	Supplementation with *L. reuteri* enhanced early growth performance, stimulated immune responses, improved ileal morphology, and reduced *Salmonella* counts by 0.2 log CFU/g in broiler chickens	Salim et al. (2013)
*L. reuteri* S5	*L. reuteri* S5 @ 1.5 × 10^8 CFU/mL oral/day for 14 days	Enteritidis	1–14 days; 1 × 10^9 CFU/mL orally	Cages; Broilers	*L. reuteri* S5 increased the relative abundance of Lactobacillaceae, decreased Enterobacteriaceae, reduced intestinal permeability, and significantly lowered cecal *Salmonella* counts by 0.51 log CFU/g	Shi et al. (2022)
*Lactobacillus plantarum* CWBI‐B659 (P)	*L. plantarum* CWBI‐B659 (P) @ 1 × 10^9 CFU/kg feed	Typhimurium	3 days; 1 × 10^8 CFU/bird orally	Cages; Ross male broilers	*L. plantarum* probiotic strain and an exogenous xylanase added to a wheat‐based diet for broiler chickens were partly expressed by FCR improvement and by a significant reduction of *Salmonella* Typhimurium population in excreta by 0.04 log CFU/g	Vandeplas et al. (2009)
*Pediococcus acidilactici*	0.1 g/kg (0.01%) feed of probiotic blend containing *P. acidilactici*	Typhimurium	3 days; 0.5 mL of 1 × 10^5 CFU/mL/bird orally	Pens; Ross 308 broilers	Supplementation of *P. acidilactici s*ignificantly reduced *Salmonella* population by 0.81 log CFU/g and to some extent was efficacious in combatting the negative effects of the challenge on broiler performance and gut health	Jazi et al. (2018)
*C. butyricum*	*C. butyricum* @ 1 × 10^7 CFU/kg feed	No challenge	N/A	Cages; Lingnan Yellow male broilers	Supplementation of *C. butyricum* did not impact on body weight gain, whereas it decreased serum immunoglobulins (IgA and IgG) and significantly reduced *Salmonella* population by 0.74 log CFU/g in ceca	Yang et al. (2012)
*C. butyricum*	*C. butyricum* @ 2 × 10^7 CFU/kg feed	No challenge	N/A	Cages; Lingnan Yellow male broilers	Supplementation of *C. butyricum* improved growth performance. In addition, it decreased serum immunoglobulins (IgA and IgG) and significantly reduced *Salmonella* population by 1.26 log CFU/g in ceca	Yang et al. (2012)
*C. butyricum*	*C. butyricum* @ 3 × 10^7 CFU/kg feed	No challenge	N/A	Cages; Lingnan Yellow male broilers	Supplementation of *C. butyricum* improved growth performance. In addition, it decreased serum immunoglobulins (IgA and IgG) and significantly reduced *Salmonella* population by 1.40 log CFU/g in ceca	Yang et al. (2012)
*E. faecium*	*E. faecium* NCIMB 11181 @ 4 × 10^8 CFU/kg of feed	Typhimurium	10 and 11 days; 1 mL 1 × 10^9 CFU/mL orally	Cages; Arbor Acor AA+ male broilers	Dietary *E. faecium* NCIBM 11181 supplementation significantly reduced *Salmonella* population by 1.53 log CFU/g. Additionally, *E. faecium* NCIMB 11181 significantly blocked *Salmonella* intestinal colonization and translocation, and reduced gut morphological structure damage and intestinal cell apoptosis by increasing gut villous height and reducing intestinal TUNEL‐positive cell numbers	Shao et al. (2022)
*Bacillus velezensis* CE 100	*B. velezensis* CE 100 @ 1 × 10^5 CFU/mL and product added 10 g/kg (1%) of feed	No challenge	N/A	Pens; Ross 308 broilers	Supplementation of *B. velezensis* CE improved litter quality and reduced the incidence of foot pad dermatitis, whereas the addition of *B. velezensis* CE 100 reduced *Salmonella* population by 0.61 log CFU/g in the intestine	Park and Sun (2022)
*S. cerevisiae*	*S. cerevisiae* @ 2 × 10^12 CFU/kg feed	No challenge	N/A	Pens; Ross 308 broilers	Supplementation of *S. cerevisiae* improved apparent ileal digestibility of nutrients, leading to improved production performance and carcass traits, and in addition, it significantly reduced *Salmonella* population by 0.7 log CFU/g	Khalid et al. (2021)

Abbreviation: DFM, direct fed microbial.

The distinction between challenge and natural‐exposure studies is material to interpretation. Challenge models establish a high, synchronized, and uniform colonization pressure that maximizes the measurable effect size of an intervention, whereas natural exposure in commercial flocks is lower, asynchronous, and heterogeneous between birds. Log reductions from challenge studies should therefore be regarded as upper‐bound estimates of the effect achievable under commercial conditions, and this qualification applies to the majority of the studies summarized in Tables 2, 3, 4, 5, 6.

**TABLE 3:** Prebiotic‐based strategies evaluated for controlling *Salmonella* colonization in broiler chickens, with or without *Salmonella* challenge.

Prebiotics	Prebiotic dose	*Salmonella* serotype	Challenge day and dose	Rearing condition and broiler type	Major findings	References
Fructo‐oligosaccharides (FOS)	9.9 g/kg (0.99%) fructo‐oligosaccharides (FOS) in feed	Typhimurium	1 day; 0.5 mL of 10^4 CFU/bird orally	Pens; Cobb broilers	Supplementation with FOS alone did not affect growth performance but reduced cecal *Salmonella* levels by 0.78 log CFU/g	Telg and Caldwell (2009)
Fructo‐oligosaccharides (FOS)	3 g/kg (0.3%) fructo‐oligosaccharides (FOS) in feed	No challenge	N/A	Cages; Arbor Acres broilers	FOS supplementation improved feed conversion ratio and intestinal microflora balance by increasing *Lactobacillus* and reducing cecal *Salmonella* by 0.97 log CFU/g	Li et al. (2008)
Xylo oligosaccharides (XOS)	2 g/kg (0.2%) active Xylo oligosaccharides (XOS) in feed	Typhimurium	3 days; 0.5 mL of 1 × 10^6 CFU/mL/bird orally	Pens; Ross 308 broilers	Supplementation with XOS significantly reduced *Salmonella* colonization, internal organ invasion, heterophil‐to‐lymphocyte ratio and improved intestinal mucosal morphology, with *Salmonella* levels decreasing by 0.39 log CFU/g on Day 10 and 0.25 log CFU/g on Day 24	
Wheat bran 280 µm particle size	10 g/kg (1%) of 280 µm size wheat bran in feed	Enteritidis	10 days; 10^8 CFU per bird orally	Pens; Ross 308 broilers	Reduced particle size of wheat bran enhanced cecal fermentation, increasing SCFA—particularly butyric and propionic acids—which downregulated *Salmonella* invasion genes in epithelial cells and significantly reduced *Salmonella* by 2 log CFU/g	Vermeulen et al. (2017)
Wheat bran 1690 µm particle size	10 g/kg (1%) of 1690 µm size wheat bran in feed	Enteritidis	10 days; 10^8 CFU per bird orally, seeder	Pens; Ross 308 broilers	Reduced particle size of wheat bran (1690 µm) did not efficiently enhance SCFA production through cecal fermentation yet still reduced *Salmonella* population by 0.5 log CFU/g	Vermeulen et al. (2017)
Trehalose	50 g/kg (5%) trehalose in feed	Typhimurium	28 days; 3.5 × 10^8 CFU/mL/bird orally	Pens; Arbor Acres male broilers	Supplementation with 5% trehalose significantly downregulated the Toll‐like receptor 4‐mediated NFκB pathway and proinflammatory cytokines such as IL1β, while upregulating anti‐inflammatory cytokines IL10 and interferon α; it also reduced AST and ALT levels, markers of liver damage, increased *Lactobacillus* abundance, and reduced *Salmonella* by 0.86 log CFU/g	Wu et al. (2020)
Gum Arabica	10 g/kg (1%) Gum Arabica in feed	No challenge	N/A	Cages; Ross 308 male broilers	Supplementation with Gum Arabica increased protein and HDL levels, decreased cholesterol, LDL, and creatinine, significantly reduced *Salmonella* by 0.9 log CFU/g, and improved gut integrity by increasing villus length and crypt depth ratio	Al‐Baadani et al. (2022)
Red Seaweed	2 g/kg (0.2%) red seaweed in feed	No challenge	N/A	Cages; Ross 308 broilers	Supplementation with red seaweed enhanced villus height and width, increased NH_3_ and H_2_S emissions, reduced *Salmonella* by 0.07 log CFU/g, and improved meat quality traits by decreasing drip loss, water‐holding capacity, and cooking loss	Balasubrama nian et al. (2021)
Asparagus	50 g/kg (5%) trimmed asparagus byproduct (TABP) in feed	No challenge	N/A	Cages; Ross 308 broilers	Supplementation with TABP decreased the atherogenic and thrombogenicity indices of meat, increased feed intake, villus height in the duodenum and jejunum, crypt depth in the jejunum and ileum, elevated acetic, propionic, butyric, and total VFA levels, and significantly reduced *Salmonella* by 0.58 log CFU/g	Nopparatmaitree et al. (2022)

Abbreviation: SCFA, short‐chain fatty acid.

**TABLE 4:** Organic acid‐based strategies evaluated for controlling *Salmonella* colonization in broiler chickens, with or without *Salmonella* challenge.

Organic acid	Organic acid dose	*Salmonella* serotype	Challenge day and dose	Rearing condition and broiler type	Major findings	References
Mix organic acids (fumaric, citric, malic and MCFAs: capric and caprylic)	0.5 g/kg (0.05%) coated organic acid with 17% fumaric acid, 13% citric acid, 10% malic acid, and 1.2% MCFAs (capric and caprylic acid, provided as a 1:1 mixture product) in feed	No challenge	N/A	Pens; Ross 308 unsexed broilers	Supplementation with an organic acids mix improved body weight gain, feed conversion ratio, and dry matter digestibility, increased *Lactobacillus* population, reduced *Salmonella* by 0.02 log CFU/g without affecting carcass quality, and lowered ammonia levels	Nguyen and Kim (2020)
Mix organic acids (fumaric, citric, malic and MCFAs: capric and caprylic)	0.75 g/kg (0.075%) coated organic acid with 17% fumaric acid, 13% citric acid, 10% malic acid, and 1.2% MCFAs (capric and caprylic acid, provided as a 1:1 mixture product) in feed	No challenge	N/A	Pens; Ross 308 unsexed broilers	Supplementation with an organic acids mix improved body weight gain, feed conversion ratio, and dry matter digestibility, increased *Lactobacillus* population, reduced *Salmonella* by 0.06 log CFU/g without affecting carcass quality, and decreased ammonia levels	Nguyen and Kim (2020)
Mix organic acids (fumaric, citric, malic and MCFAs: capric and caprylic)	1 g/kg (0.1%) coated organic acid with 17% fumaric acid, 13% citric acid, 10% malic acid, and 1.2% MCFAs (capric and caprylic acid, provided as a 1:1 mixture product) in feed	No challenge	N/A	Pens; Ross 308 unsexed broilers	Supplementation with an organic acids mix improved body weight gain, feed conversion ratio, and dry matter digestibility, increased *Lactobacillus* population, reduced *Salmonella* by 0.09 log CFU/g without affecting carcass quality, and decreased ammonia levels	Nguyen and Kim (2020)
Mix organic acids (propionic, formic, and HMTBa)	0.2 g/kg (0.02%) organic acids mixture of propionic acid, formic acid and HMTBa in feed	No challenge	N/A	Pens, open‐sided house; Cobb 500 broilers	Supplementation with an organic acids mix significantly increased body weight gain, apparent ileal digestibility, and apparent metabolizable energy, enhanced *Lactobacillus* population, and reduced *Salmonella* by 0.16 log CFU/g	Islam et al. (2022)
Mix organic acids and salt encapsulated in vegetable oil	0.6 g/kg (0.06%) of Galliacid containing organic acids and salts encapsulated in oil in feed	No challenge	N/A	Pens; Ross 308 unsexed broilers	Supplementation with 0.06% Galliacid increased body weight gain and feed conversion ratio and significantly reduced *Salmonella* population by 1.801 log CFU/g	Hassan et al. (2010)
Mix organic acids, salt, and flavoring agent encapsulated in vegetable oil	1 g/kg (0.1%) of Biacid containing organic acids and salts encapsulated in oil in feed	No challenge	N/A	Pens; Ross 308 unsexed broilers	Supplementation with 0.1% Biacid increased body weight gain and feed conversion ratio and significantly reduced *Salmonella* population by 1.297 log CFU/g	Hassan et al. (2010)
Formic acid	5 mL/L (0.5%) formic acid in drinker water	Typhimurium	35 and 41 days; 1 × 10^8 CFU/mL/bird orally	Pens; broilers	Supplementation of formic acid in drinking water before feed withdrawal significantly reduced *Salmonella* population by 0.6 log CFU/g	Byrd et al. (2001)
Acetic acid	5 mL/L (0.5%) acetic acid in drinker water	Typhimurium	35 and 41 days; 1 × 10^8 CFU/mL/bird orally	Pens; Broilers	Supplementation of acetic acid in drinking water before feed withdrawal significantly reduced *Salmonella* population by 0.63 log CFU/g	Byrd et al. (2001)
Butyric acid	5 g/kg (0.5%) butyric acid in feed	Typhimurium	3 days; 0.5 mL of 1 × 10^5 CFU/mL/bird orally	Pens; Ross 308 male broilers	Supplementation with butyric acid significantly reduced *Salmonella* Enteritidis population in the ceca by 1.35 log CFU/g at Day 7 post‐infection	Jazi et al. (2018)
Lactic acid	5 mL/L (0.5%) lactic acid in drinker water	Typhimurium	35 and 41 days; 1 × 10^8 CFU/mL/bird orally	Pens; Broilers	Supplementation of lactic acid in drinking water before feed withdrawal significantly reduced *Salmonella* population by 0.4 log CFU/g	Byrd et al. (2001)
Ascorbic acid	0.1 g/kg (0.01%) ascorbic acid in feed	Enteritidis	6 days; 1 × 10^7 CFU/bird orally	Cages; Cobb male broilers	Supplementation with ascorbic acid reduced *Salmonella* by 0.32 log CFU/g in the prophylactic model and significantly by 4.72 log CFU/g in the therapeutic model; additionally, it significantly decreased total intestinal IgA levels	Hernandez‐Patlan et al. (2019)
Caprylic acid	10 g/kg (1%) caprylic acid in feed from Days 37 to 42	Enteritidis	25 days; 1 × 10^8 CFU/mL/bird orally	Pens; Cornish cross broilers	Supplementation with caprylic acid significantly reduced *Salmonella* population by approximately 3 log CFU/g	Johny et al. (2009)
Calcium formate	0.5 g/kg (0.05%) calcium formate	No challenge	N/A	Pens; Cobb 400 mixed‐sex broilers	Supplementation increased villus height without affecting serum lipid profile, including total cholesterol, HDL cholesterol, and LDL cholesterol, and significantly reduced *Salmonella* population by 1.78 log CFU/g	Pathak et al. (2016)

**TABLE 5:** Essential oil‐based strategies evaluated for controlling *Salmonella* colonization in broiler chickens, with or without *Salmonella* challenge.

Essential oil	Essential oil dose	*Salmonella* serotype	Challenge day and dose	Rearing condition and broiler type	Major findings	References
*Trans*‐cinnamaldehyde + Eugenol	10 g/kg (1%) eugenol (EG) given from Days 37 to 42 in feed	Enteritidis	30 days; 1 × 10^8 CFU/mL/bird orally	Pens; Ross broilers	Supplementation with EG had no adverse effects on the liver and significantly reduced *Salmonella* colonization in the ceca by 1.5 log CFU/g	Kollanoor‐Johny et al. (2012)
*Trans*‐cinnamaldehyde + Eugenol	7.5 g/kg (0.75%) mix of trans‐cinnamaldehyde (TC) given from Days 37 to 42 in feed	Enteritidis	30 days; 1 × 10^8 CFU/mL/bird orally	Pens; Ross broilers	Supplementation with TC had no adverse effects on the liver and significantly reduced *Salmonella* colonization in the ceca by 1.5 log CFU/g	Kollanoor‐Johny et al. (2012)
Cinnamaldehyde + thymol	0.05 g/kg (0.005%) cinnamaldehyde and thymol in feed	Enteritidis	7 days; 2.83 × 10^8 CFU/mL/bird orally	Pens; Hubbard male broilers	Supplementation with an essential oil mix reduced *Salmonella* prevalence by 31.3%, although the concentration used increased fecal *Salmonella* populations by 1.09 log CFU/g	Cerisuelo et al. (2014)
Cinnamaldehyde + thymol	0.1 g/kg (0.01%) cinnamaldehyde and thymol in feed	Enteritidis	7 days; 2.83 × 10^8 CFU/mL/bird orally	Pens; Hubbard male broilers	Supplementation with an essential oil mix reduced *Salmonella* prevalence by 25% and decreased fecal *Salmonella* population by 0.77 log CFU/g	Cerisuelo et al. (2014)
Carvacrol, thymol, and paracymene mix	300 µL carvacrol, thymol, and paracymene mix essential oils daily orally for 14 days	No challenge	N/A	Cages; Ross 308 mixed sex broilers	Supplementation with an essential oil mix significantly increased sucrase and lactase activity in the intestinal mucosa, enhanced the expression of occludin, claudin‐1, claudin‐5, ZO‐1, and ZO‐2 genes, elevated goblet cell content in the small intestine epithelium, and significantly reduced *Salmonella* population by 0.71 log CFU/g	Liu et al. (2018)
Carvacrol, thymol, and paracymene mix	400 µL carvacrol, thymol, and paracymene mix essential oils daily orally for 14 days	No challenge	N/A	Cages; Ross 308 mixed sex broilers	Supplementation with an essential oil mix significantly increased sucrase and lactase activity in the intestinal mucosa, upregulated the expression of occludin, claudin‐1, claudin‐5, ZO‐1, and ZO‐2 genes, enhanced goblet cell content in the small intestine epithelium, and significantly reduced *Salmonella* population by 0.94 log CFU/g	Liu et al. (2018)
Microencapsulated thymol	1 mL/L (0.1%) microencapsulated thymol oil in drinking water from 5 DPC for 3 days	Enteritidis	15 days; 1 × 10^9 CFU/mL/bird orally	Pens; Cobb broilers	Treatment with thyme microemulsion and thyme oil significantly reduced *Salmonella* Enteritidis population in the ceca by up to 2.5 log CFU/g, lowered mortality rates, improved tissue integrity, and accelerated antibiotic residue clearance from broiler organs	Hamed et al. (2022)
Microencapsulated oil mix from oregano, rosemary, cinnamon, and chili pepper	0.15 g/kg (0.015%) microencapsulated photo essential oil mix from oregano, rosemary, cinnamon, and chili pepper extract in feed	No challenge	N/A	Pens, open‐sided house; Cobb 500 broilers	Supplementation with an essential oil mix increased lymphoid organ weight, improved apparent ileal digestibility and apparent metabolizable energy, enhanced *Lactobacillus* population, and reduced *Salmonella* by 0.17 log CFU/g	Islam et al. (2022)
Microencapsulated oil mix from thyme, savory, peppermint, and black pepper	1 g/kg (0.1%) microencapsulated essential oil mix from thyme, savory, peppermint, and black pepper in feed	Enteritidis	3 days; 1 × 10^9 CFU/mL/bird orally	Pens; Ross 308 male broilers	Supplementation with an essential oil mix increased villus height, villus width, villus‐to‐crypt ratio, and goblet cell numbers, decreased crypt depth, and significantly reduced *Salmonella* population in the ceca by 0.68 log CFU/g	Moharreri et al. (2022)
Microencapsulated oil mix from thyme, savory, peppermint, and black pepper	2 g/kg (0.2%) microencapsulated essential oil mix from thyme, savory, peppermint, and black pepper in feed	Enteritidis	3 days; 1 × 10^9 CFU/mL/bird orally	Pens; Ross 308 male broilers	Supplementation with an essential oil mix increased villus height, villus width, villus‐to‐crypt ratio, and goblet cell numbers, decreased crypt depth, and significantly reduced *Salmonella* population by 1.02 log CFU/g	Moharreri et al. (2022)
Coriander oil	25 µL/L (0.0025%) coriander essential oil nanoemulsion in drinking water	No challenge	N/A	Pens; New Lohman male broilers	Supplementation with coriander oil improved body weight gain, feed conversion ratio, and increased villus height, width, and crypt depth; it also enhanced lactic acid bacteria (LAB) populations and significantly reduced *Salmonella* by 4.9 log CFU/g	Sholiha et al. (2023)
Curcumin	0.1 g/kg (0.01%) curcumin in feed	Enteritidis	6 days; 1 × 10^7 CFU/bird orally	Cages; Cobb male broilers	Supplementation with curcumin reduced total intestinal IgA levels and significantly decreased *Salmonella* population by 1.69 log CFU/g	Hernandez‐Patlan et al. (2019)

**TABLE 6:** Bacteriophage‐based strategies evaluated for controlling *Salmonella* colonization in broiler chickens, with *Salmonella* challenge.

Bacteriophage	Bacteriophage dose	*Salmonella* serotype	Challenge day and dose	Rearing condition and broiler type	Major findings	References
SPFM10–SPFM14 (cocktail)	0.1×, 1×, and 10× doses via feed (10^5^, 10^6^, and 10^7^ PFU/day/bird)	Typhimurium (SL1344)	Day 4; 10^6^ CFU/bird orally	Pens; Ross 308 broilers; 7 birds/pen; 16 pens/group	All phage doses significantly reduced *Salmonella* colonization. The 0.1× dose (10^5^ PFU/day) completely cleared *Salmonella* from feces and ceca by Day 42. The 1× dose (10^6^ PFU/day) reduced cecal counts to 2.08 × 10^3^ CFU/g. The 10× dose (10^7^ PFU/day) resulted in improved performance (weight gain and feed intake), with no *Salmonella* in ceca and detection in only 2/16 pens. No phage resistance observed	Thanki et al. (2023)
Wild‐type lytic bacteriophage	2.9 × 10^10^ PFU/bird from Days 6 to 10 and 6.8 × 10^10^ PFU/bird from Days 31 to 35, both given orally for 5 consecutive days	Enteritidis	1 day; 6 × 10^6 CFU/mL/bird orally	Cages; Embrapa 021 broilers	Early phage therapy (Days 6–10) reduced *Salmonella* Enteritidis counts in the cecum by 2.4 log_10_ CFU/g at Day 20 (10 days posttreatment), showing statistically significant reduction compared to controls. Late phage therapy (Days 31–35) similarly reduced cecal *S*. Enteritidis by 2.5 log CFU/g at Day 40. Both treatments were effective in reducing intestinal colonization, with no adverse effects observed in birds. Phages were recovered from ceca, confirming gastrointestinal survival	Vaz et al. (2020)
Bacteriophage *Φ*151 (*Salmonella enterica* Enteritidis P125109)	1 mL PBS containing 30% (wt/vol) CaCO_3_ containing 1 × 10^11 PFU/mL/bird on Day 38 orally	Enteritidis	36 days; 1 × 10^8 CFU/mL/bird orally	Pens; Ross broiler	High‐dose *Φ*151 treatment significantly reduced *S*. Enteritidis cecal colonization by 2.52 log CFU/g after 24 h. Phage resistance emerged but was not stable in vitro or in vivo	Atterbury et al. (2007)
Bacteriophage *Φ*10 (*S. enterica* Typhimurium 4/74)	1 mL PBS containing 30% (wt/vol) CaCO_3_ containing 1 × 10^11 PFU/mL/bird Day 38 orally	Typhimurium	36 days; 1 × 10^8 CFU/mL/bird orally	Pens; Ross broiler	High‐dose *Φ*10 treatment significantly reduced *Salmonella* Typhimurium cecal colonization by 2.19 log CFU/g after 24 h. Phage resistance emerged rapidly but was transient in most isolates	Atterbury et al. (2007)
Liposome encapsulated bacteriophages UAB_*Φ*20, *Φ*78, and *Φ*87	0.1 mL of 10^11 PFU/mL/bird from Days 1–7 orally	Typhimurium	1 day; 0.1 mL of 1 × 10^8 CFU/mL/bird orally	Pens; Ross 308 broilers	Liposome‐encapsulated phages showed significantly improved gastric stability and intestinal retention. They reduced cecal *Salmonella* by up to 3.9 log CFU/g and provided protection up to 1 week posttreatment, unlike nonencapsulated phages, whose effect declined after 72 h	Colom et al. (2015)
Bacteriophage P1:1	10^10 PFU/kg feed	Enteritidis	1 day; 10^4 CFU/bird orally	Cages; Broilers	No statistically significant reduction in *S*. Enteritidis recovery from ceca compared to controls. Phage P1:1 had no effect on colonization or recovery	Sklar and Joerger (2001)
Bacteriophage (EP2 + MUT3 + M4 + YP)	2 × 10^12/kg feed	Enteritidis	1 day; 10^8 CFU/bird orally	Cages; Broilers	Phage cocktail resulted in a modest but statistically significant reduction (about 1 log CFU/g) of *S*. Enteritidis in ceca by Days 3 and 5 post‐challenge	Sklar and Joerger (2001)

#### 6.2.2 Prebiotics

Prebiotics are indigestible feed components that traverse the upper gastrointestinal tract (GIT) and are selectively fermented by beneficial microorganisms, enhancing their activity (Micciche et al. 2018). These compounds resist absorption in the upper GIT, withstand acidic conditions, promote beneficial microbial growth, improve metabolic function, and modulate the host immune system (Sarao and Arora 2017). Common prebiotics in poultry production include fructo‐oligosaccharides (FOS), inulin, mannan‐oligosaccharides (MOS), and xylo‐oligosaccharides (XOS), many of which are derived from yeast cell walls and fermentation by‐products (Jahan et al. 2022).

Selective fermentation reflects enzymatic differences between commensal bacteria and *Salmonella*. FOS, inulin, and XOS are preferentially utilized by beneficial taxa such as *Bifidobacterium*, some *Lactobacillus*, and xylanolytic *Bacteroides*, which produce inhibitory metabolites, including SCFAs (Gibson et al. 2017). In contrast, *Salmonella* has limited capacity to directly degrade many complex prebiotic oligosaccharides, although cross‐feeding may allow utilization of smaller fragments released by other microbiota members. MOS acts mainly through a non‐fermentative mechanism, in which mannose residues interfere with Type 1 fimbriae‐mediated attachment of *Salmonella* to intestinal receptors, promoting clearance with digesta (Spring et al. 2000).

By stimulating beneficial bacteria (*Bacteroides* and *Bifidobacterium)*, prebiotics enhance host health and restrict colonization of foodborne pathogens (Ricke 2021). This modulation of gut microbiota and fermentation processes disrupts pathogen establishments (Johnson et al. 2015; Lee et al. 2002). Prebiotics, rich in fiber and oligosaccharides, also augment amylase production in the GIT, improving broilers growth rates (Xu et al. 2003). Certain prebiotics provide binding sites that facilitate bacterial clearance from the digestive tract, preventing *Salmonella* adhesion to intestinal epithelial cells (Donalson et al. 2008). Furthermore, multiple studies have documented reductions in *Salmonella* populations, particularly during hen molting, through elevated concentrations of SCFAs (Cummings and Macfarlane 2002; Durant et al. 2000), which can be accomplished through prebiotic administration (Donalson et al. 2008; Ricke 2015). FOS supplementation showed varied effects: at 0.99%, it had no impact on growth but reduced cecal *Salmonella* by 0.78 log CFU/g (Telg and Caldwell 2009), whereas at 0.3%, it improved feed conversion and microbial balance, lowering *Salmonella* by 0.97 log CFU/g (Li et al. 2008). XOS at 0.2% significantly reduced *Salmonella* colonization, internal invasion, and improved gut morphology, with reductions of 0.39 and 0.25 log CFU/g on Days 10 and 24, respectively (Jazi et al. 2019; Table 3). The 0.2% inclusion reported by Jazi et al. (2019) is an experimental rather than a commercial dose. Typical commercial XOS inclusion in broiler diets is an order of magnitude lower, in the range of 0.01%–0.05% (100–500 g/t), a level set primarily by ingredient cost rather than by dose‐optimization trials. The dose–response relationship is incompletely characterized and appears not to be monotonic: Increases in beneficial cecal taxa and in SCFA concentration have been reported across roughly 0.005%–0.02% inclusion, whereas higher levels have in some studies produced no additional benefit or a reduction in performance, plausibly because rapidly fermentable oligosaccharide reaching the caecum in excess alters osmotic load and shifts fermentation toward less desirable end products (De Maesschalck et al. 2015).

#### 6.2.3 Organic Acids

Organic acid supplementation in poultry feed or water has become a cornerstone in ABF poultry production systems. These additives include short‐chain fatty acids (C ≤ 4) such as formic, acetic, propionic, and butyric acids, as well as medium‐chain fatty acids (C6–C12) like caproic, caprylic, capric, and lauric acids. Along with other organic acids like lactic acid, they work by acidifying the feed and the bird’s gastrointestinal tract, which ultimately suppresses pathogens and supports gut health. (Dittoe et al. 2018). Organic acids likely exert their antibacterial effects by lowering feed and gut pH, which disrupts crop and proventriculus physiology, creating an inhospitable environment for pathogens (Van Immerseel et al. 2006; Wang et al. 2025). Depending on pH, they can transition between undissociated and dissociated forms, facilitating their antimicrobial action by permeating through microbial membranes (Huyghebaert et al. 2011). Once inside the cell, weak acids dissociate, inhibiting bacterial enzymes and nutrient transport systems (Huyghebaert et al. 2011). Byrd et al. (2001) suggested that adding lactic acid to drinking water before transport could potentially decrease *Salmonella* and *Campylobacter* contamination in the bird crop and consequently in broiler carcasses during processing. Similarly, Heres et al. (2004) showed that highly acidified feed rapidly reduced *Campylobacter* and *Salmonella* levels in vitro, and acidified feed reduced the susceptibility to *Campylobacter* colonization but not *Salmonella*. The efficacy of organic acids produced by probiotics in reducing foodborne pathogens such as *Campylobacter* and *Salmonella* in poultry is evident (Dittoe et al. 2018; Neal‐Mckinney et al. 2012).

Medium‐chain fatty acids exhibit greater efficacy against *Salmonella* than short‐chain variants, but butyrate (a SCFA) demonstrated the strongest bactericidal activity against *Escherichia coli* and *Salmonella* (Van Immerseel et al. 2006; Khan and Iqbal 2016). Medium‐chain fatty acids are generally more potent than SCFAs because their greater lipophilicity allows them to partition more readily into bacterial membranes. Like other organic acids, they act mainly in the undissociated form, enter the bacterial cell, dissociate in the near‐neutral cytoplasm, and disrupt intracellular pH, osmotic balance, and enzyme function. However, medium‐chain fatty acids, particularly C6–C12, can also insert into the lipid membrane, increasing permeability and causing leakage of ions and ATP, which enhances bactericidal activity (Desbois and Smith 2010). They may also interfere with *Salmonella* virulence signaling and reduce SPI‐1‐mediated invasion. In contrast, butyrate acts mainly through transcriptional repression of SPI‐1 rather than direct killing (Gantois et al. 2006). The efficacy of medium‐chain fatty acids in vivo depends on delivery, and protected or microencapsulated forms are often more effective because they survive the upper GIT and reach the lower intestine and ceca, where *Salmonella* colonization occurs.

Supplementation with organic acids via feed or water showed varying degrees of *Salmonella* reduction in broilers. Formic, acetic, and lactic acids (each at 0.5% in drinking water) administered before feed withdrawal reduced *Salmonella* by 0.6, 0.63, and 0.4 log CFU/g, respectively (Byrd et al. 2001). Butyric acid at 0.5% in feed reduced cecal *S*. Enteritidis by 1.35 log CFU/g (Jazi et al. 2018), whereas caprylic acid at 1% achieved a 3 log CFU/g reduction (Johny et al. 2009). Ascorbic acid reduced *Salmonella* by 0.32 log CFU/g prophylactically and by 4.72 log CFU/g therapeutically, along with a decrease in intestinal IgA levels (Hernandez‐Patlan et al. 2019; Table 4). Therefore, by reducing gut *Salmonella* populations, organic acids mitigate carcass contamination during processing, aligning with ABF goals. Pre‐transport water acidification may reduce crop *Salmonella* contamination, but its efficacy depends on bird consumption; therefore, low‐pH water should be used cautiously because reduced intake during feed withdrawal may worsen dehydration, live shrinkage, and yield loss.

A reduction of 4.72 log CFU/g would be of considerable practical significance if reproducible under commercial conditions. Quantitative risk assessments consistently show that human illness risk from poultry is driven by concentration rather than prevalence, and reductions of this magnitude in cecal load would translate into a substantially lower probability of carcass contamination exceeding the thresholds associated with illness, exceeding what is typically achieved by any single postharvest antimicrobial intervention (Oscar 2021).

#### 6.2.4 Essential Oils or Botanicals

Essential oils are volatile plant‐derived compounds composed of terpenoids and low molecular weight hydrocarbons, including thymol, carvacrol, eugenol, cinnamaldehyde, and phenols (Jamroz et al. 2006). Moreover, terms, phytogenic feed additives, phytonutrients, or phytobiotics, botanicals encompass diverse bioactive plant compounds, including phenolic compounds (such as apigenin, quercetin, curcumin, and resveratrol), organosulfur compounds (such as allicin), terpenes (including eugenol, thymol, carvacrol, and artemisinin), and aldehydes (such as cinnamaldehyde and vanillin) (Rossi et al. 2020). Essential oils are known to interact with microbial lipid bilayers due to their hydrophobic nature, causing cell lysis and content leakage (O'bryan et al. 2015). Additionally, they inhibit biofilm formation and pathogenicity and interfere with bacterial communication (quorum sensing), a process that regulates gene expression via population‐dependent signaling molecules (Maurya et al. 2021). Essential oils promote gut health and often enhance the microbial community, consequently, reduce the colonization of foodborne pathogens. The effectiveness of these oils varies based on the inclusion level and feed composition. Although their antimicrobial mechanisms are not fully understood, several potential antimicrobial outcomes may indirectly affect *Campylobacter* and *Salmonella*. Essential oils improve ileal absorption of amino acids in broilers (Jamroz et al. 2006), thus limiting a necessary nutrient source for *Campylobacter* in the ceca (Velayudhan et al. 2004). Essential oils may enhance the immune response and reduce foodborne pathogens concentrations in the poultry gut (Diaz‐Sanchez et al. 2015). Supplementation with various essential oils significantly reduced *Salmonella* populations in broilers. Eugenol at 1% reduced *S*. Enteritidis colonization in the ceca by 1.5 log CFU/g (Kollanoor‐Johny et al. 2012), a carvacrol–thymol–paracymene mix reduced *Salmonella* by 0.94 log CFU/g (Liu et al. 2018), and a microencapsulated blend of thyme, savory, peppermint, and black pepper reduced *Salmonella* by 1.02 log CFU/g (Moharreri et al. 2022; Table 5).

Essential oil carry‐over is a valid commercial concern, especially for lipophilic phenolic compounds such as thymol and carvacrol, which have low sensory detection thresholds. However, this concern depends strongly on dose and application method. At typical in‐feed levels used in broiler diets, approximately 50–300 mg/kg, thymol and carvacrol generally do not impair cooked meat flavor, odor, or overall acceptability; some studies report improved sensory quality due to reduced lipid oxidation and lower thiobarbituric acid reactive substances during storage (Botsoglou et al. 2002). In contrast, direct postharvest application of essential oils to carcasses or meat products poses greater risk of off‐flavor and has limited commercial adoption despite strong antimicrobial activity. Encapsulation, nanoemulsions, and edible coatings may reduce volatile sensory carry‐over while maintaining antimicrobial efficacy (Donsi and Ferrari 2016).

#### 6.2.5 Bacteriophages

Bacteriophages (phages) are viruses that specifically infect and lyse bacterial cells. Upon attaching to susceptible bacteria, bacteriophages replicate via two cycles: lytic (virulent and productive) or lysogenic (temperate and dormant) (Żbikowska et al. 2020). During the lytic cycle, the phage injects genetic material into the host cell, generating viral components, and lyses the host cell to release progeny phages. In the lysogenic cycle, phage DNA integrates into the host genome or persists episomally, replicating passively without immediate cell lysis. Most of the bacteriophages used to control foodborne pathogens are lytic phages. Bacteriophages are being employed as antibiotic replacements due to their remarkable specificity in targeting pathogens, reduced risk of allergic reactions, and lack of detrimental effects on the host normal gut microbiota (Liang et al. 2023; Shaji et al. 2023). Lytic phages reduce *S*. Enteritidis prevalence in broiler ceca (Kimminau et al. 2020) and alleviate clinical symptoms (e.g., diarrhea and appetite loss) in challenged birds (Nabil et al. 2018). Phage therapy was also shown to decrease *Salmonella* Typhimurium colonization in internal organs and enhance chick survival (Hao et al. 2023). However, oral phage treatment alone did not restore the intestinal dysbiosis (dysbiosis is an imbalance or maladaptation of the microbial communities, particularly in the gut, that can disrupt normal physiological functions and lead to various health issues) of intestinal flora in infected chicks (Hao et al. 2023). *Salmonella* phages obtained from a broiler farm and wastewater treatment facilities showed strong lytic activity (85.7%–96.4%) against more than 56 *Salmonella* serovars (Pelyuntha et al. 2022). Feeding broilers a phage cocktail (SPFM10–SPFM14) at doses of 10^5^–10^7^ PFU/day significantly reduced *S*. Typhimurium colonization. The lowest dose completely cleared *Salmonella* by Day 42, and no phage resistance emerged (Thanki et al. 2023; Table 6). Similarly, phage cocktails reduce *Campylobacter* by 1–1.5 log in commercial broilers (Wagenaar et al. 2005; Fischer et al. 2013). A constraint of phage therapy is its narrow host range, as phages can only lyse their specific hosts. Similarly, successful phage therapy relies on adsorption to bacterial cells, which may be hindered within the digesta matrix (Greer 2005). Additionally, the emergence of phage resistance (bacteria can develop resistance to phages over time) is another disadvantage (Luong et al. 2020). Furthermore, phages do not resolve dysbiosis caused by infections (Hao et al. 2023). Despite limitations, phage targets specific pathogens without harming gut microbiota (Liang et al. 2023; Shaji et al. 2023), and there are no reported allergic reactions.

#### 6.2.6 Bacteriocins

Bacteriocins are antimicrobial peptides produced by certain bacteria, notably lactic acid bacteria (*Lactobacillus*, *Lactococcus*, and *Pediococcus*), which are often used in probiotics for their dual role in pathogen inhibition via bacteriocin production and compounds like lactic acid and hydrogen peroxide (Darbandi et al. 2022; Simons et al. 2020). Although it is extensively studied in swine, its application in poultry remains underexplored (Thippareddi and Singh 2022). Nisin‐containing formulations decreased *S*. Typhimurium and spoilage microorganism populations on poultry products (Natrajan and Sheldon 2000). The bacteriocins produced by probiotics *Ligilactobacillus salivarius* UO.C109 and *Ligilactobacillus saerimneri* UO.C121 shifted the bacterial community, enhanced diversity, and reduced pathogens (e.g., Enterobacteriaceae) in *Salmonella* inoculated simulated chicken gut environment (Miri et al. 2023). Enterocin E‐760, derived from *Enterococcus* sp. NRRL B‐30745, effectively reduced the *Campylobacter* colonization in market‐age broilers when administered through their feed (Line et al. 2008). Albusin B, a bacteriocin produced by *Ruminococcus albus* 7, was shown to enhance nutrient absorption, elevate *Lactobacillus*, and reduce *Salmonella* population in feces. This leads to improved performance in broiler chickens (Wang et al. 2011). Although bacteriocin‐producing strains offer cost benefits, scalable production requires strain optimization (Ben Lagha et al. 2017).

### 6.3 Vaccines

Vaccines are biological preparations (live‐attenuated, inactivated, or subunit) that stimulate the host immunity against target microorganisms and neutralize them when encountered (Marouf et al. 2022). In poultry, three *Salmonella* vaccine types are employed. Live‐attenuated vaccines mimic natural infection by adhering to the intestinal mucosa upon oral administration, triggering robust humoral and cell‐mediated immune responses (Jia et al. 2020). These vaccines are effective in breeders to confer offspring immunity, though broilers’ immature immunity limits their efficacy (Van Immerseel et al. 2005). Thus, it is ideal for vaccinating breeders to confer protective immunity to the broilers. Vaccination is a preferred method widely adopted in the layer industry for controlling *S*. Enteritidis within layer flocks to reduce the risk of *S*. Enteritidis contamination in shell eggs. The administration of an *S*. Enteritidis Δ*hilAssrAfliG* mutant strain to 1‐day‐old broiler chickens led to a notable decrease in fecal shedding and colonization, in contrast to exposure to a virulent *S*. Enteritidis strain (De Cort et al. 2013). Killed *Salmonella* vaccines are serovar‐specific, induce humoral immunity, and require boosters (Deguchi et al. 2009). Despite the limited cell‐mediated immunity elicited, killed vaccines are favored over live vaccines due to considerations of biosecurity and safety (Acevedo‐Villanueva, Akerele, et al. 2021). Furthermore, multivalent inactivated vaccines are essential for comprehensive protection to combat the diverse array of *Salmonella* serotypes prevalent in poultry populations (Huberman et al. 2022; Marouf et al. 2022). Subunit vaccines containing defined antigens are considered safer than live‐attenuated or inactivated vaccines (Kang et al. 2022). Vaccines utilizing outer membrane proteins and flagella proteins of *Salmonella*, along with adjuvants, have effectively reduced bacterial shedding and induced a robust antigen‐specific immune response in layers chicken (Renu et al. 2020). Nanoparticles and phospholipid–calcium cochleates have also been investigated as vaccine delivery systems in poultry (Acevedo‐Villanueva, Akerele, et al. 2021). The chitosan nano vaccine exhibited no detrimental effect on the performance of broiler chickens. Additionally, it successfully triggered a targeted immune response against *Salmonella* and shows promise in diminishing *Salmonella* cecal colonization in broiler birds (Acevedo‐Villanueva et al. 2020). When administered in ovo, the chitosan nano vaccine induced an antigen‐specific immune response against *Salmonella* and decreased *Salmonella* cecal load in broilers (Acevedo‐Villanueva, Akerele, et al. 2021; Table 7). Experimental advancements in vaccine approaches show promising prospects for enhancing both efficacy and coverage.

**TABLE 7:** Vaccine‐based strategies evaluated for controlling *Salmonella* colonization in broiler chickens, with *Salmonella* challenge.

Vaccine	Vaccine dose	*Salmonella* serotype	Challenge day and dose	Rearing condition and broiler type	Major findings	References
*Salmonella* Enteritidis CPH (recombinant)	1.6 × 10^7 CFU/bird (estimated) on Day 1 as spray	Heidelberg	1.6 × 10^7^ CFU/bird (estimated) on Day 1 as spray	Pens; Broilers	Spray vaccination with SE‐CPH significantly reduced cecal colonization of *Salmonella* Heidelberg in broilers. At 4 days post‐challenge (Day 18), vaccinated birds had 2.75 log CFU/g compared to 5.60 log CFU/g in controls. At 11 days post‐challenge (Day 25), levels were 1.89 vs. 3.98 log CFU/g	Yang et al. (2017)
Chitosan nanoparticle (SE OMP FP) vaccine	40 µg/bird gel‐spray on Day 1 + 20 µg/bird oral booster on Day 7	Enteritidis	14 days; 1 × 10^7 CFU/bird orally	Pens; Cobb broilers	Vaccinated birds showed a 2‐log CFU/g reduction in cecal and spleen *S*. Enteritidis, and 0.6‐log_10_ in small intestine at 21 dpi	Acevedo‐Villanueva et al. (2022)
Chitosan nanoparticle (SE OMP FP) vaccine	1 mg (0.2 mL) in amniotic cavity on ED 18	Enteritidis	7 days; 1 × 10^9 CFU/bird orally	Pens; Cobb broilers	3‐log CFU/g reduction in cecal *S*. Enteritidis at 7 DPC (Day 14); significantly elevated bile IgA and serum IgY levels. Nanoparticle delivery enhanced mucosal and systemic immunity (*p* < 0.05)	Acevedo‐Villanueva, Renu, et al. (2021)
*S*. Enteritidis Chitosan nanoparticle + CpG adjuvant (in ovo)	1 mg CNP + CpG on ED 18 (in ovo)	Enteritidis	7 days; 1 × 10^9^ CFU/bird orally	Pens; Cobb broilers	CpG addition enhanced Th1‐type response (↑IFN‐γ), reduced *Salmonella* recovery by around 2.8 log CFU/g compared to control. Induced both humoral and cellular responses with improved cytokine balance (*p* < 0.05)	Acevedo‐Villanueva, Renu, et al. (2021)
*S*. Typhimurium PBAD‐mviN recombinant	1 × 10^8^ CFU orally on Days 2 and 7	Typhimurium	Day 14; 2 × 10^8^ CFU/bird orally	Cages; Cobb 500 male broilers	Reduced cecal colonization by 2.7 log CFU/g at 42 dpi compared to unvaccinated controls. Induced protective immunity and lowered bacterial recovery significantly (*p* < 0.05)	Rubinelli et al. (2015)

The emergence of multidrug‐resistant strains necessitates the development of more effective vaccines. Recent advancements in genetic engineering have enabled the tailoring of *Salmonella* strains with specific attenuating mutations, such as *aroA* mutant *S*. Typhimurium, improving their safety and immunogenicity (Jia et al. 2020). Additionally, understanding the role of host immune responses, particularly Th1‐type immunity, is crucial for vaccine development (Mastroeni and Ménager 2003). Although significant progress has been made, multidrug‐resistant strains necessitate novel vaccines, emphasizing ongoing research into genetic and immune mechanisms.

## 7 *Salmonella* Contamination in the Poultry Hatchery

In integrated broiler operations, *Salmonella* transmission can be traced from breeder farms to hatcheries, highlighting an endemic challenge requiring comprehensive biosecurity across all production stages (Kim et al. 2007). Hatcheries serve as critical hubs, consolidating eggs from multiple flocks and distributing chicks to farms, but they also exhibit higher *Salmonella* detection rates compared to production farms due to biosecurity gaps (Rothrock et al. 2024; Adhikari et al. 2024). Key contamination pathways include (a) contaminated eggs, emphasizing the need for stringent biosecurity (Oastler et al. 2022) and (b) hatchery environment, including air, water, and facility surfaces, which facilitates *Salmonella* dissemination. Floor drains and transport trucks are identified as critical hot spots due to high *Salmonella* prevalence (Rothrock et al. 2024; Figure 6). Additionally, chick handling, hatcher, and waste handling areas are more contaminated compared to setter areas, indicating a need for targeted cleaning and disinfection in these zones (Oastler et al. 2022). Equipment used in the hatchery, including egg picking cups, hatcher baskets, harbor, and spread *Salmonella* from contaminated eggs to fresh eggs (Arvanitoyannis et al. 2009). Improper cleaning and disinfection of the dirty hatcher and hatcher baskets can create fomites that further disseminate the *Salmonella* to workers, clothes, and equipment in the hatcher, perpetuating environmental persistence. Environmental samples, such as hallway fans, fluff samples, have confirmed the presence of motile, multidrug resistance *Salmonella* complicating control efforts (Rothrock et al. 2024; Zamil et al. 2021).

**FIGURE 6:**
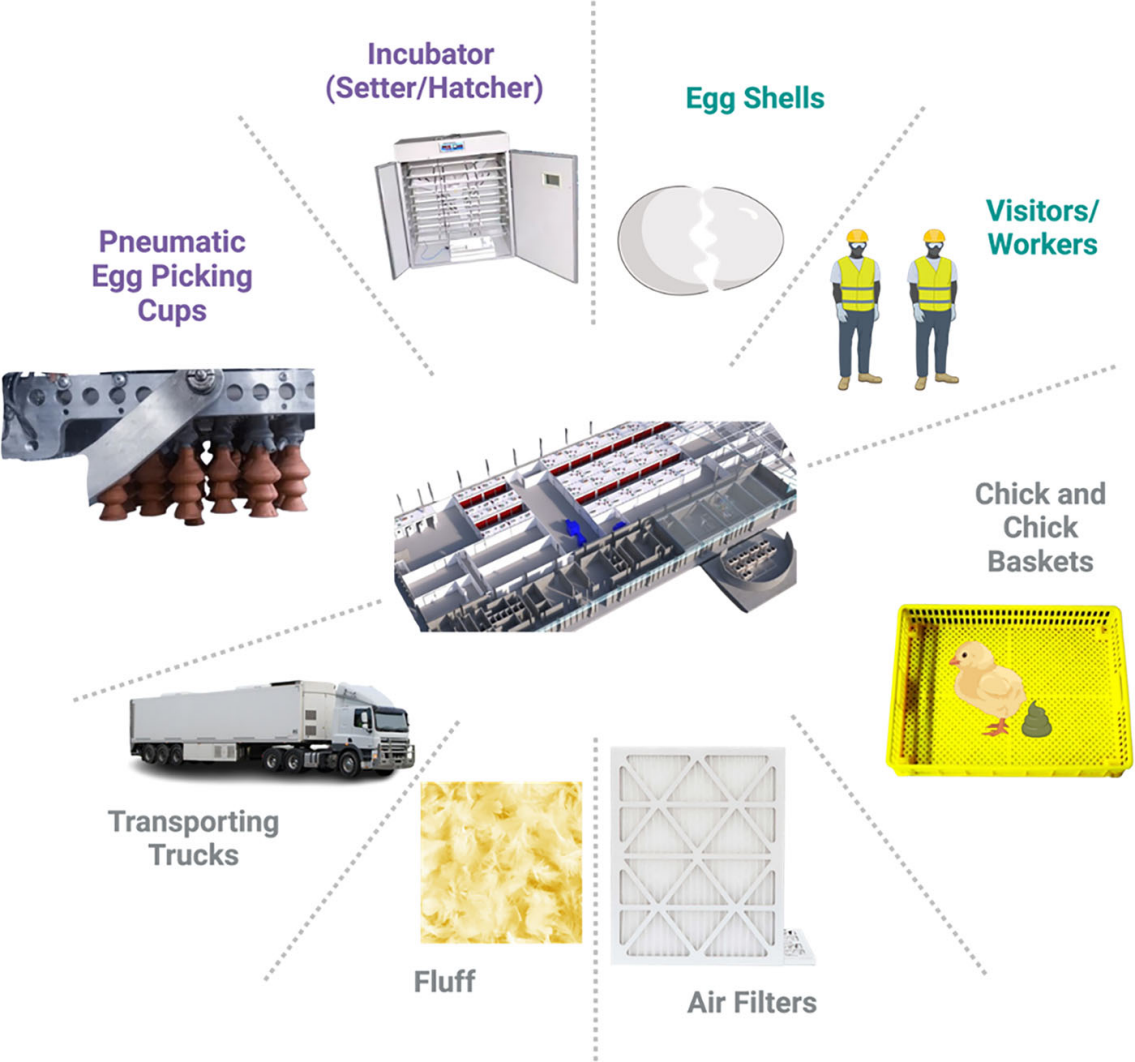
Potential sources of *Salmonella* contamination in broiler hatchery.

## 8 Control of *Salmonella* in Poultry Hatchery

A vertically integrated approach to *Salmonella* spanning all production stages, including hatcheries, is critical for a safer and more sustainable poultry industry. Although past decades have prioritized *Salmonella* control in processing plants, hatchery environment remained unstudied due to limited regulations, detection methods, and industry focus. Addressing *Salmonella* contamination early in production can reduce downstream prevalence, making hatcheries a strategic control point. However, effective interventions require a comprehensive understanding of hatchery microbiota.

Effective control measures involve bio‐mapping, ensuring robust biosecurity practices, and implementing cleaning and disinfection. Bio‐mapping is the practice of sampling at specific places in processing plants where contamination levels are assessed (Singh and Thippareddi 2024). However, to understand the ecology of *Salmonella* within hatcheries, several bio‐mapping studies are needed, and one study revealed *Salmonella* prevalence up to 26%, in areas like floor drains and transport trucks showing even higher contamination levels. This knowledge enables targeted cleaning and disinfection efforts, optimizing sanitation protocols. Regular bio‐mapping also allows the effectiveness of control measures to be monitored and making necessary adjustments. By utilizing bio‐mapping, hatcheries can reduce *Salmonella* contamination and improve the overall hygiene and biosecurity of their facilities.

A strategically planned hatchery layout with clearly demarcated clean, neutral, and dirty zones is essential to prevent pathogen dissemination (Mauldin 2002; Lister 2008). To further improve biosecurity, color‐coded personal protective apparel can visually delineate movement between various zones. Moreover, implementing a unidirectional flow for eggs, such as from storage rooms and clean incubators to hatcher trays and dispatching rooms, can reduce the risk of cross‐contamination (Thermote 2006; Hafez 2007; Woodger and Wirral 2009).

Cleaning and disinfection are other important aspects of controlling *Salmonella* in the hatchery. Over the last decade, toxic formaldehyde has been replaced with alternatives such as quaternary ammonium compounds, peroxidase‐catalyzed compounds, and garlic‐based sanitizers that maintain hatchability and reduce microbial contamination (Gonzalez et al. 2022; Kim and Kim 2010; Oliveira et al. 2021, 2022). Additionally, surfaces that come into contact with eggs and personnel require disinfection with appropriate disinfectants. Although many disinfectants demonstrate efficacy against *Salmonella* in vitro, their effectiveness can be compromised in real‐world hatchery conditions due to organic matter and high microbial loads (Møretrø et al. 2012). For best practice, the industry should prioritize rapid‐acting agents, thorough staff training, and optimized application methods to overcome time constraints between hatch cycles.

## 9 Sources of *Salmonella* Contamination in Poultry Processing Plant

*Salmonella* contamination in poultry processing plants poses a significant public health risk, primarily through the transmission of the pathogen to consumers. The USDA‐FSIS regularly monitors *Salmonella* prevalence in poultry processing facilities and evaluates them according to established performance standards (USDA FSIS 2021). *Salmonella* is ubiquitous in poultry production, and several interrelated factors influence its presence in the preharvest environment (Newell and Fearnley 2003). Contamination of poultry carcasses with *Salmonella* seems mostly linked to flock contamination during rearing and transportation to slaughter (Newell et al. 2011). Birds are typically transported to the processing plant in coops that are not routinely cleaned and sanitized between uses, potentially spreading microorganisms from one flock of birds to another (Slader et al. 2002). The high incoming load of pathogen populations poses a challenge for poultry processors in reducing and eliminating *Salmonella* and *Campylobacter* in poultry meat (Slader et al. 2002). Broiler processing comprises all the operations from the arrival of the birds at the processing plant to the time the product leaves the plant (Figure 7). From receiving live birds to finished products, interventions are incorporated to reduce and eliminate foodborne pathogens and ensure food safety (NACMCF 2023). This includes proper handling, hygienic slaughter, evisceration, effective chilling, and thorough sanitation to mitigate the risk of *Salmonella* and *Campylobacter* contamination (Andreoletti et al. 2010).

**FIGURE 7:**
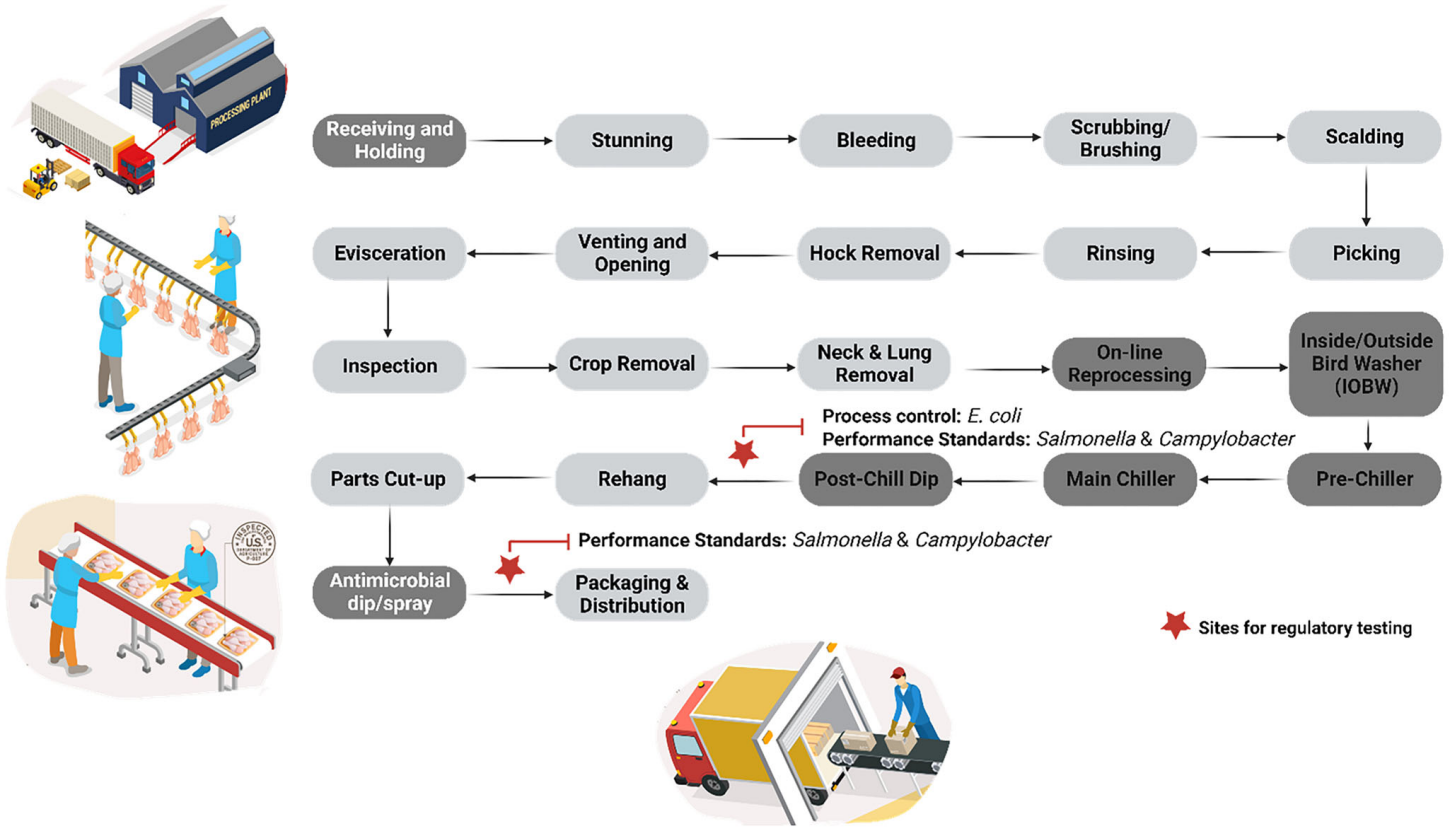
The diagram illustrates the sequential stages in commercial poultry processing, beginning with live bird receiving and continuing through stunning, scalding, defeathering, evisceration, chilling, and deboning, leading to final packaging. Adopted from Vaddu (2024).

### 9.1 Feed Withdrawal and Transportation Stress

Restricting access to feed for the birds before transportation and processing reduces the amount of excreta during transport to processing facilities and diminishes the risk of cross‐contamination (Hinton et al. 2000). However, prolonged feed withdrawal can reduce the strength of the intestines and increase the probability of gut breakage during processing. Transporting broilers to processing plants can increase the prevalence of *Salmonella* and *Campylobacter* on the broilers due to cross‐contamination from the excreta of neighboring birds (Slader et al. 2002; Newell et al. 2011). The contact between broilers and the excreta on the floor of the coop facilitates the transfer of pathogens onto their skin and feathers (Newell et al. 2011). Transportation‐related stress increases levels of corticosterone and other stress‐related hormones, which affect GIT function (Jacobs‐Reitsma 1997). This increases the excreta inside the transport crates and the risk of cross‐contamination (Slader et al. 2002).

### 9.2 Stunning

Electric stunning (ES) renders the bird unconscious before exsanguination. The commonly used ES method involves dipping the birds’ head into a saline solution while passing an electric current to cause unconsciousness (Harris 2018). During controlled atmosphere stunning and killing (CASK), birds are killed before shackling through exposure to a gradual, multiphasic change in the atmosphere, often with an increasing concentration of carbon dioxide (CO_2_) to induce death. Higher stress and injury rates associated with ES may increase the risk of pathogen contamination (Shields and Raj 2010). Although stunning is not directly associated with increased *Salmonella* contamination, it can create conditions conducive to bacterial spread. Specifically, electrical or gas stunning methods may induce regurgitation or fecal release from birds, potentially contaminating nearby carcasses or equipment (Harris 2018).

### 9.3 Exsanguination

Exsanguination or bleeding is an essential part of poultry processing. It is achieved by severing the jugular veins and carotid arteries using a rotating circular blade, causing a rapid loss of blood and, ultimately, death (Bolder 2007; Plumber et al. 2012). Birds are bled for 1–2 min, which is essential to maintain meat quality (Plumber et al. 2012). Proper bleeding prevents blood from collecting in bird tissues, which lessens discoloration and improves the overall appearance of meat (Plumber et al. 2012). Dark patches might result from residual blood in veins and arteries or in tiny blood vessels under the skin, resulting in an unappealing appearance (Davis and Coe 1954). Studies show that improper handling and hygiene during exsanguination significantly increase the risk of *Salmonella* contamination. However, improved sanitation practices during and immediately after this stage can effectively prevent leakage and cross‐contamination of carcasses (Althaus et al. 2017).

### 9.4 Scalding

Hot water immersion breaks down the proteins that bind the feathers to the skin and facilitates picking by loosening the feather follicles (USDA‐FSIS 2021). Although scalding is an essential step in picking, it also marks a step in the processing line where carcasses are exposed to a shared bath, which may lead to cross‐contamination (Zhuang et al. 2013). The line speed or immersion time must be controlled to ensure that each bird is adequately exposed to the scalding temperature to avoid over‐scalding that causes skin damage, affecting the final product's visual and textural quality (Buhr et al. 2003). For this purpose, soft scalding or hard scalding is used. Soft scalding for broilers requires 90–120 s exposure time in water temperatures between 51°C and 54°C (USDA‐FSIS 2021). The cuticle of the skin remains intact, preserving the epidermis and yellow appearance of the carcass, making this method ideal for processing fresh carcasses and marketing them as whole birds (Shung et al. 2022). Hard scalding for broilers requires a higher water temperature of 59–64°C with a shorter exposure duration of 30–75 s (USDA‐FSIS 2021). In the US, most commercial broiler processing operations primarily utilize hard scalding to improve picking efficacy, facilitating better adherence to batter and breading during fast food processing (Shung et al. 2022). To ensure effective scalding, water quality, temperature, and proper agitation of water are recommended (USDA‐FSIS 2021). A counter‐flow pattern of the carcasses and the clean water in the scald tank is essential to minimize cross‐contamination and promote proper heat exchange (Cason et al. 2004). Impurities are pushed away from cleaner carcasses by adding clean water at the exit of the scalding tank and letting it flow toward the entrance (Buhr et al. 2014). Compressed air constantly agitated water to ensure uniform heat distribution and minimize cold spots (Zhuang et al. 2013). Precise water temperature control is essential to avoid over‐scalding of the skin or a low temperature to increase the difficulty of feather picking (Harris 2018).

### 9.5 Picking

Picking, also called defeathering, is an essential stage in the processing of chickens, where the feathers and outer layer of skin are removed from the carcasses (USDA‐FSIS 2021). Carcasses are passed through rubber fingers that mechanically remove the loosened feathers (Berrang and Dickens 2004). Proper process control is essential as picking is a point where cross‐contamination can occur via water and contact with contaminated rubber‐picking fingers (Geornaras et al. 1997). Picking can introduce fecal contamination, while agitation and contact between carcasses can promote cross‐contamination (Buhr et al. 2003). Several factors contribute to higher microbial populations associated with mechanical picking methods. Antimicrobials, such as hydrogen peroxide, acetic acid, and chlorine, could be used as interventions during picking to reduce the pathogen and control cross‐contamination (Kumar et al. 2020). Poultry processors can improve product safety by lowering the danger of cross‐contamination, keeping an eye on equipment, and employing antimicrobial interventions (Harris 2018).

### 9.6 Evisceration

The evisceration process in broiler processing is a critical stage for controlling cross‐contamination. Each step presents specific risks for microbial contamination, necessitating rigorous control measures to assure food safety. Initially, rehanging involves transferring carcasses from the picking line to the evisceration line. This step is susceptible to cross‐contamination due to handling multiple carcasses, which can spread microorganisms from one bird to another if sanitation protocols are not strictly followed (Berghaus et al. 2022). Oil gland cutting near the tail area can introduce contaminants if not executed cleanly, as the gland is often laden with bacteria from the skin and feathers (Kumar et al. 2022). During venting, an incision is made around the vent to allow for organ removal, and any accidental puncturing of the intestines can result in fecal contamination of the carcass (Rosenquist et al. 2006). The opening requires precision to avoid cutting into the intestines, and automated machinery typically handles evisceration and viscera removal, but any lapses in equipment cleaning can propagate bacteria across multiple carcasses (Arsenault et al. 2007). Crop removal involves extracting the crop from the digestive system. Improper handling can cause spillage of its contents, which are often rich in bacteria. Subsequent steps, such as neck breaking and neck skinning, also require careful handling to prevent contamination from the respiratory tract (Kumar et al. 2022). Lung removal must be performed with attention to hygiene to avoid spreading respiratory pathogens (USDA‐FSIS 2021). Inside–Outside Bird Washers (IOBWs) are important for cleaning carcasses, both inside and outside, but they need regular maintenance and sanitation to prevent becoming sources of contamination themselves (USDA‐FSIS 2021). These steps within the evisceration process underscore the necessity for stringent hygiene practices and meticulous control measures to minimize the risks of pathogens, thereby ensuring the safety and quality of broiler meat.

### 9.7 Chilling

In poultry processing plants, the chilling stage plays an important role in ensuring food safety and extending shelf life by reducing microbial populations. This method in poultry processing is a critical step aimed at rapidly reducing the temperature of processed birds to inhibit microbial growth and maintain meat quality. There are two primary methods for chilling broilers: immersion chilling and air chilling, each favored in different regions (USDA FSIS 2021). Immersion chilling, predominant in the United States, involves submerging carcasses in chilled water (0–4°C). This method facilitates rapid cooling due to counter‐current flow, where carcasses and water move in opposite directions (USDA FSIS 2021). However, immersion‐chilling can lead to water absorption, with carcasses retaining moisture through subsequent processing stages (Young and Smith 2004). The exchange of contaminated water among carcasses acts as a medium for pathogens like *Salmonella* to spread from contaminated to uncontaminated carcasses, necessitating stringent water hygiene management throughout the chilling process (Leone et al. 2024). Furthermore, improperly maintained water temperatures or circulation rates during immersion chilling can create environments favorable for *Salmonella* persistence (Harris 2018). Studies demonstrate that such conditions allow *Salmonella* to survive, emphasizing the importance of rigorous temperature and flow monitoring (Bucher et al. 2012). Another critical concern is the formation of biofilms on the surfaces of chilling tanks and equipment. Biofilms provide a protective environment for pathogens such as *Salmonella*, making them resistant to standard cleaning protocols and acting as reservoirs for ongoing contamination (Xiao et al. 2019). Poor hygienic practices, such as insufficient washing or mishandling of carcasses before immersion, also exacerbate contamination risks. Ensuring proper pre‐chilling sanitation measures is essential for mitigating these risks (Buncic and Sofos 2012). A specific challenge in the immersion chilling process is the resilience of *S*. Enteritidis, known for its ability to survive at low temperatures. This pathogen's resistance to the chilling process underscores the need for targeted antimicrobial interventions during poultry processing (Nagel et al. 2013).

### 9.8 Poultry Parts

After the carcasses are sufficiently cooled to the target temperature of 4°C, they are sent to the next processing stage for, deboning, and further processing (Lillard 1971). During this stage of processing, several methods are employed to transform chilled carcasses into a variety of chicken products. First, the carcasses undergo partitioning, where they are divided into major components such as thighs, wings, breasts, and drumsticks, catering to diverse consumer preferences for different chicken cuts (Kingsbury et al. 2023). Throughout the process, there is a potential microbial contamination risk associated with handling and cutting carcasses. Cross‐contamination is more likely while cutting as it entails direct physical contact with equipment (Rivera‐Pérez et al. 2014). The risk is further increased by handling and interaction between several carcasses, underscoring the importance of strict sanitation and hygiene procedures (Powell et al. 2012). Strict safety and quality control procedures are used in broiler processing facilities to reduce the risk of contamination. This entails routine equipment cleaning, careful handling procedures, chemical interventions, and contamination source monitoring (Kumar et al. 2020; Perez‐Arnedo et al. 2021).

## 10 *Salmonella* Serotypes Found on Broiler Carcasses in Processing Plant

*S. enterica*, a species with over 2600 diverse serotypes, exhibits complex dynamics during poultry processing (Rasamsetti and Shariat 2023). Studies reveal that multiple serotypes often coexist within a single *Salmonella*‐positive carcass sample and can also be present within a single broiler flock at the time of processing (Ramirez‐Hernandez et al. 2019; Rasamsetti et al. 2022). These serotypes often differ significantly from those isolated directly on farms due to the processing environment, contamination pathways, and the cumulative effects of transport and holding conditions (Newell et al. 2011). The rehang stage, a pivotal point in poultry processing where carcasses transition into the plant after defeathering, is often associated with elevated contamination levels resulting from handling and transportation conditions (Rasamsetti and Shariat 2023). Notably, recent research highlights the increasing prevalence and persistence of *Salmonella* Infantis in poultry processing environments (Siceloff et al. 2022), suggesting its tolerance to antimicrobials used during processing. Furthermore, some *S*. Infantis serotypes exhibit multidrug resistance, amplifying concerns over their control (Zeng et al. 2021). Studies by Berrang et al. (2007) emphasized its association with contamination originating from transport crates and slaughter equipment, compounded by contact with processing machinery. The pre‐chill stage is another important control point because carcasses may reflect contamination acquired during earlier processing steps such as scalding, defeathering, and evisceration. Rivera‐Pérez et al. (2014) identified multiple slaughter‐line contamination risk points, whereas Boubendir et al. (2021) reported that *Salmonella* serotypes such as Kentucky were among the most frequently recovered during processing and highlighted chilling as a critical control step. These findings emphasize the importance of effective sanitation of water systems and processing equipment to limit cross‐contamination. Antimicrobial interventions applied during the post‐chill stage aim to reduce microbial contamination. Despite these measures, certain serotypes exhibit notable resilience. *Salmonella* Kentucky, while effectively reduced during post‐chill, as noted by Golden and Mishra (2020), continues to be detected, albeit at significantly lower levels. In contrast, *S*. Typhimurium and *S*. Infantis persist, demonstrating tolerance to interventions such as peroxyacetic acid (PAA), as highlighted in the same study.

## 11 Control Strategies of *Salmonella* in the Processing Plant

*Salmonella* control in poultry processing plants involves a range of strategic interventions to minimize bacterial contamination and enhance food safety. The USDA‐FSIS mandates the HACCP system, which establishes performance standards for *Salmonella* (Obe et al. 2020). These standards require that fewer than 9.8% of poultry carcasses sampled test positive, with plants achieving “Category 1” status maintaining contamination rates at or below half of this limit. These standards are essential for guiding antimicrobial interventions and maintaining low pathogen levels throughout processing stages (Obe et al. 2023). Advanced control measures within the plant include using antimicrobials such as peroxyacetic acid and sodium hypochlorite, which are applied at critical stages to reduce *Salmonella* prevalence. Studies employing microbial bio‐mapping highlight the effectiveness of quantifying bacterial load at various processing points, such as pre‐ and post‐chill stages, to optimize antimicrobial applications where they are most needed, thereby enhancing intervention precision and efficacy (De Villena et al. 2022; Rasamsetti and Shariat 2023). However, some *Salmonella* serotypes, including Enteritidis and Infantis, have demonstrated a degree of tolerance to specific antimicrobials, which complicates control efforts in processing environments. Biosecurity practices within the plant, such as controlling rodent populations, minimizing dust, and enforcing strict sanitation protocols, are crucial to supplement antimicrobial treatments and reduce cross‐contamination risks (Obe et al. 2023). Protective measures, such as using designated clothing and footwear for plant personnel, prevent pathogens from spreading across processing areas. Additionally, peroxyacetic acid and other acid‐based sanitizers applied during chilling stages have shown particular efficacy in lowering *Salmonella* loads, as these stages are critical control points for ensuring pathogen reduction before product distribution (Rasamsetti and Shariat 2023). Reducing the population of *Salmonella* and cross‐contamination in processing operations requires strict adherence to sanitation and hygiene regulations. The microbial burden may be significantly decreased by thoroughly and regularly cleaning the facilities and equipment used in processing facilities (Loretz et al. 2010).

### 11.1 Antimicrobial Interventions in Processing Plant

Antimicrobial interventions in poultry processing plants are essential to ensure poultry products’ microbial safety and quality. These interventions are designed to reduce or eliminate pathogenic bacteria *Salmonella*, a major concern in the poultry industry (USDA FSIS 2021). One of the most critical stages where chemical treatments are applied is in the chillers, where broiler carcasses are cooled down rapidly after evisceration (Leone et al. 2024; Table 8).

**TABLE 8:** Key antimicrobial interventions in poultry processing plants.

Intervention	Mechanism	Concentration (ppm)	Efficacy (log reduction)
Chlorine	Oxidative damage, membrane disruption	≤50	1–2
Peroxyacetic acid (PAA)	Oxidation, pH disruption	≤2000	1.5–2.5
Cetylpyridinium chloride	Membrane disruption	≤8000	∼1.7

*Source*: USDA‐FSIS (2021) and Vaddu et al. (2021).

#### 11.1.1 Inorganic and Organic Chlorine‐Based Treatments

Within the poultry processing line, chlorine is commonly used as an antimicrobial in IOBWs, pre‐chillers, main chillers, post‐chill treatments, and other stages due to its low concentration requirements for efficacy and relatively low cost (Dogan et al. 2022). Chlorine has been the main antimicrobial used in the United States for chilling poultry carcasses; up to 50 ppm of free available chlorine is permissible (Singh et al. 2020). However, several factors significantly impact its efficacy. Even with the constant addition of chlorine to the chiller, the organic and inorganic material in the cooling water reduces the available chlorine (Simpson and Mitch 2022). Furthermore, the effectiveness of chlorine as an antibacterial is significantly influenced by the pH level of the water (Kere Kemp et al. 2001). Changes influence chlorine efficiency in pH due to altering their chemical forms. When pH levels increase, the accessible hypochlorous acid (HOCl) in the solution converts into hypochlorite ions (ClO^−^). This conversion occurs because the chemical equilibrium between hypochlorous acid and hypochlorite ions is pH‐dependent (Simpson and Mitch 2022). Therefore, although the increase in pH may enhance the production of hypochlorite ions, which still possess some antibacterial activity, the overall efficacy of chlorine as a disinfectant decreases in alkaline conditions due to the reduced presence of the more potent hypochlorous acid form.

#### 11.1.2 Peroxyacetic Acid (PAA)

Peroxyacetic acid (PAA) is more commonly used as an antimicrobial to reduce the prevalence and concentration of pathogens (Singh et al. 2020). Hydrogen peroxide, acetic acid, and peroxy compound combined to create an organic oxidant PAA. Because it can be employed in a wide range of temperatures (0–40°C) and pH levels (3–7.5), PAA is especially recognized for its adaptability to different processing settings such as spray or immersion application (Cheng et al. 2020). In contrast to chlorine, PAA remains effective even in the presence of organic materials, such as proteins and fats (Je et al. 2023). Chlorine has long been the preferred antimicrobial for pathogen control in poultry processing. However, the efficacy of PAA at pH values above 7.0 is not affected, which is more advantageous for yield gain (Leone et al. 2024). Many chicken processors have switched from using chlorine to this more effective solution at neutral and slightly alkaline pH levels. PAA is approved for use in raw poultry products at a maximum concentration of 2000 ppm (USDA FSIS 2026). Studies have shown a significant reduction in pathogen population upon exposure for a short duration (<30 s), like the dip‐tank employed in post‐chill interventions for whole carcasses or parts (Rincon et al. 2020; Vaddu et al. 2021). PAA can present occupational health and safety issues notwithstanding its antimicrobial properties. PAA can irritate skin, mucous membranes, and eyes and be corrosive to equipment (Kataria et al. 2020). With proper application, PAA remains an effective antimicrobial intervention in chicken processing despite these difficulties (Kataria et al. 2020). Peroxyacetic acid is a useful chemical intervention that has benefits over other antimicrobials, such as chlorine. However, because the other antimicrobials are underrepresented, there is no firm evidence of the relative efficacy of any of the antimicrobials (Ebel et al. 2019). Poultry processors find it a desirable alternative due to its broad range of effectiveness and capacity to sustain antimicrobial activity even in the presence of organic materials. Vaddu et al. (2021) demonstrated that immersion of chicken wings in PAA solutions at 100 ppm for 60 min can significantly reduce *Salmonella* populations by up to 1.78 log CFU/mL, regardless of the solution's pH, showcasing its robust antimicrobial efficacy. This effectiveness is attributed to PAA's combined acidic and oxidizing properties, which eliminate pathogens without generating harmful by‐products, making it a safer alternative to chlorine. Given the continued predominance of *Salmonella* serotypes such as *S*. Infantis in poultry production environments, it is critical to better understand the role of PAA in resistance dynamics to support sustainable antimicrobial practices and effective pathogen control throughout the food supply chain (Chapagain et al. 2026).

Peroxyacetic acid (PAA) has traditionally been considered low risk for resistance development because its oxidative activity damages multiple cellular targets, including thiol groups, membrane lipids, and nucleic acids. However, repeated or sublethal exposure can induce adaptive tolerance in *Salmonella* through oxidative stress responses involving OxyR, SoxRS, catalase, alkyl hydroperoxide reductase, and RpoS‐mediated general stress pathways (Chapagain et al. 2026). This is particularly relevant where organic load, uneven spray coverage, or shielded carcass sites reduce effective PAA concentration.

PAA tolerance may also be serotype‐dependent, with *S*. Typhimurium and *S*. Infantis showing greater tolerance than *S*. Kentucky under comparable conditions. Because oxidative stress pathways overlap with broader antimicrobial stress responses, sublethal PAA exposure may contribute to co‐selection for reduced susceptibility to other antimicrobials, although evidence for clinically relevant antibiotic cross‐resistance remains limited (Obe et al. 2023). Practical mitigation should focus on maintaining validated PAA concentrations at the point of carcass contact, avoiding prolonged low‐dose exposure, sequencing antimicrobials with different modes of action, and including PAA‐tolerance monitoring in plant verification programs. Standardized definitions and testing protocols for PAA tolerance remain an important research need.

#### 11.1.3 Cetylpyridinium Chloride

CPC is a quaternary ammonium compound widely utilized as an antimicrobial in poultry processing plants, particularly in the post‐chill phase. CPC's cationic nature interacts with negatively charged phospholipids in bacterial cell membranes, increasing permeability and causing the leaking of cellular contents and ultimately cell death (Breen et al. 1997). The use of CPC in post‐chill processing ensures that the microbial load on the poultry carcasses is significantly lowered, enhancing product safety and extending the shelf life. Its efficacy in pathogen control helps in meeting regulatory standards and protecting public health by minimizing the risk of foodborne illnesses (Rincon et al. 2020). The maximum allowable concentration of CPC for surface treatment of raw poultry carcasses, giblets, or parts is 8000 ppm, whether applied by spray or immersion (USDA FSIS 2021). Immersing or spraying chicken skin with CPC at 1000 ppm resulted in an approximate 1.7 log reduction in the *Salmonella* population (Kim and Slavik 1996).

### 11.2 Biofilms and Barrier Controls

Physical separation and barrier control are critical in poultry processing plants to prevent cross‐contamination by *Salmonella*. These measures include the segregation of “clean” and “dirty” zones, ensuring that raw and processed products do not intersect (Berends et al. 1998; Olsen et al. 2003). Proper facility layout, with unidirectional flow, minimizes pathogen spread, while using smooth, easily cleanable surfaces further reduces contamination risks (Holah 2014). Maintaining and sanitizing equipment is essential, as improperly cleaned surfaces harbor pathogens and support biofilm formation (Carrasco et al. 2012).

Biofilms, formed by *Salmonella* on surfaces like stainless steel and plastic, are resilient microbial communities that resist cleaning and disinfection (Khelissa et al. 2017). Biofilms are prevalent on processing equipment, walls, and drains, serving as reservoirs for contamination. Their extracellular matrix protects bacteria from environmental stress and disinfectants, complicating eradication efforts (Davies and Breslin 2003). Physical cleaning methods, including scrubbing and high‐temperature treatments, effectively disrupt biofilm layers (Khelissa et al. 2017).

Chemical disinfectants, such as sodium hypochlorite and peroxyacetic acid, are commonly used but must be applied at appropriate concentrations and durations for success (Speranza et al. 2017). Enzymatic treatments targeting the biofilm matrix and quorum‐sensing inhibitors show promise in enhancing cleaning outcomes and preventing biofilm formation (Nahar et al. 2018). Biological agents, including probiotics and bacteriophages, inhibit biofilm formation through competitive exclusion and bacterial targeting (Gong and Jiang 2017). Gloves must be replaced and sanitized regularly to prevent pathogen transfer. Hands can serve as significant vectors for *Salmonella* if hygiene practices are inadequate (Carrasco et al. 2012). An integrated approach combining physical, chemical, and biological strategies is critical for effective *Salmonella* control in poultry processing plants. By addressing biofilm formation and ensuring rigorous hygiene and facility management, the poultry industry can significantly reduce *Salmonella* contamination and enhance food safety (Khelissa et al. 2017).

### 11.3 Good Manufacturing Practices (GMPs) and Sanitation Procedures

The control of *Salmonella* in poultry processing plants relies heavily on the implementation of robust GMPs and Sanitation Standard Operating Procedures (SSOPs). GMPs form the foundation of food safety systems, encompassing comprehensive guidelines that address hygiene, facility design, equipment maintenance, and operational procedures to mitigate microbial contamination risks (Manning et al. 2006). Personnel hygiene is a critical component of GMPs, necessitating rigorous training to ensure adherence to proper practices such as wearing sanitized uniforms, utilizing PPE, and following strict handwashing protocols to prevent cross‐contamination (Afrila et al. 2023). Facility design also plays a pivotal role in *Salmonella* control, as processing workflows must prevent contact between raw and finished products through physical separation and controlled access points, effectively reducing the risk of cross‐contamination (Loretz et al. 2010). Furthermore, regular equipment maintenance and sanitation are vital, as improperly cleaned surfaces can serve as persistent reservoirs for *Salmonella*. Validation methods such as adenosine triphosphate (ATP) bioluminescence testing ensure the efficacy of these cleaning procedures (Fries 2002). SSOPs, as part of a broader sanitation strategy, involve detailed protocols for cleaning and disinfection throughout the facility. These protocols include physical removal of debris, high‐pressure washing, and the application of chemical sanitizers such as chlorine‐based compounds, peroxyacetic acid, or quaternary ammonium compounds, which have demonstrated efficacy in eradicating *Salmonella* from surfaces (Afrila et al. 2023). Effective implementation of SSOPs requires robust monitoring and verification mechanisms, including microbial testing to detect and quantify *Salmonella* in critical control areas. Environmental monitoring programs further identify high‐risk zones, such as drains, conveyor belts, and chilling tanks, which are known to harbor persistent *Salmonella* contamination (Fries 2002). Additionally, control of secondary contamination vectors, such as aerosols and water, is critical, as these media can disseminate *Salmonella* throughout the facility if not properly managed (Afrila et al. 2023). Integrating GMPs and SSOPs provides a synergistic approach to *Salmonella* mitigation, ensuring compliance with regulatory standards such as those outlined by the USDA‐FSIS and international food safety guidelines. These practices are essential for safeguarding public health, as they effectively reduce the risk of foodborne illness associated with poultry products while maintaining consumer trust and meeting export requirements. Proactive measures, supported by ongoing research and technological advancements, remain critical in enhancing the efficacy of *Salmonella* control strategies within poultry processing plants.

A comprehensive and integrated approach is critical for effective *Salmonella* control in poultry processing facilities. This involves adhering to HACCP protocols, maintaining stringent sanitation practices, and managing environmental factors such as air and water quality (Obe et al. 2023). Facilities implementing proactive and multidimensional strategies significantly reduce *Salmonella* prevalence and enhance food safety outcomes. Addressing the persistence and adaptability of *Salmonella* requires a layered strategy combining microbial bio‐mapping, targeted antimicrobial interventions, and robust biosecurity measures (Rasamsetti and Shariat 2023). These practices mitigate contamination risks and ensure compliance with federal pathogen control standards, safeguarding public health and food safety (IFSAC 2019). Preharvest interventions, such as dietary modifications, probiotics, vaccination, and chemical treatments, play a pivotal role in reducing *Salmonella* colonization in poultry GITs (Wang et al. 2023). Postharvest measures, including antimicrobial agents like chlorine and peroxyacetic acid during critical stages such as chilling, further minimize contamination risks (Thames et al. 2022). When integrated with rigorous sanitation and hygiene protocols, these interventions create a robust defense against pathogen proliferation. Managing *Salmonella* within vertically integrated poultry systems demands a holistic framework spanning the entire production chain—from breeder and broiler houses to hatcheries and processing plants. The poultry industry can effectively disrupt pathogen transmission by unifying biosecurity measures, vaccination programs, and sanitation practices across all stages. This cohesive approach reduces *Salmonella* prevalence, ensures regulatory compliance, and delivers safer products to consumers. A steadfast commitment to these strategies strengthens food safety systems and minimizes public health risks, offering a definitive pathway to combating this pervasive pathogen.

## 12 Future Perspectives of *Salmonella* Control in Poultry Production

*Salmonella* control in vertically integrated broiler production should continue to move from reactive detection toward integrated, risk‐based prevention across breeder flocks, hatcheries, feed mills, grow‐out farms, transport, processing plants, and finished‐product verification. At the farm level, biosecurity remains essential, but future systems may increasingly incorporate automated surveillance tools, including camera‐based monitoring, vehicle‐movement tracking, environmental sensors, pest‐detection systems, and digital flock‐movement records. These tools could help identify potential contamination sources, including rodents, wild birds, personnel, equipment, vehicles, litter, and water, before they contribute to flock‐level infection.

Vaccination will remain an important preharvest control tool, particularly when targeted toward relevant *Salmonella* serotypes and integrated with breeder‐flock, hatchery, and grow‐out management. Future vaccine development should focus on broader serotype coverage, improved duration of protection, practical delivery, and compatibility with commercial broiler production. In parallel, further research is needed to identify nonantibiotic interventions that can maintain broiler performance while reducing intestinal colonization by foodborne pathogens. These may include probiotics, prebiotics, organic acids, phytogenic compounds, bacteriophages, bacteriocins, and competitive exclusion products.

Rapid detection, quantification, and genomic characterization will become increasingly important for risk‐based *Salmonella* control. Although the USDA‐FSIS raw poultry *Salmonella* framework proposed enumeration‐based and serotype‐focused approaches, the proposed rule was withdrawn in 2025 for further assessment. Therefore, these approaches should be discussed as future‐facing risk‐management tools rather than current regulatory requirements. Future control programs may benefit from stronger integration of quantitative microbial bio‐mapping, surveillance design, genomic epidemiology, predictive modeling, quantitative microbial risk assessment (QMRA), and decision‐support systems.

### 12.1 Quantitative Microbial Bio‐Mapping

Bio‐mapping complements presence/absence testing by adding quantitative microbial enumeration at defined points along the processing continuum. This approach generates a concentration profile that helps identify where microbial load increases and where interventions achieve meaningful reductions (Singh and Thippareddi 2024). Common sampling points include rehang, post‐picking, pre‐evisceration, pre‐chill, post‐chill, and parts. Hygiene indicators, including aerobic plate count, Enterobacteriaceae, and *E. coli*, may be assessed alongside *Salmonella* because *Salmonella*‐positive samples may be too infrequent for reliable process‐control charting in well‐performing plants. De Villena et al. (2022) demonstrated the value of this approach by comparing facilities operating at different antimicrobial‐intervention levels and showing that the shape of the concentration profile, rather than a single endpoint value, can indicate where intervention capacity is effective or insufficient. The practical value of bio‐mapping is that it supports intervention‐placement decisions by identifying stages with high incoming load, limited log reduction, or evidence of process drift.

### 12.2 Surveillance Design

The value of surveillance depends strongly on sampling design. Important design features include the sampling frame, sample size, sampling frequency, target serotypes, sample matrix, sampling location, and analytical method. For example, detecting 1% prevalence with 95% confidence requires approximately 300 independent samples, which is rarely feasible in routine commercial testing. Therefore, many plant‐level programs are more realistically powered to detect higher prevalence or process changes over time. Fixed‐site environmental monitoring is useful for identifying persistent contamination niches, whereas randomized product sampling is more appropriate for estimating lot‐level or shift‐level prevalence. Composite samples can improve sensitivity per unit cost, but they limit enumeration of individual units. Internal sampling programs should therefore be designed not only for regulatory verification but also to detect process drift before loss of process control or noncompliance occurs.

### 12.3 Genomic Epidemiology

Whole‐genome sequencing has become a key tool for *Salmonella* surveillance because it enables serotype prediction, antimicrobial resistance gene detection, and high‐resolution relatedness analysis in a single assay (Nadon et al. 2017). In vertically integrated poultry systems, its most important practical value is distinguishing resident strains from repeated new introductions. Repeated recovery of closely related isolates from a hatchery or processing plant over time suggests an established contamination niche requiring targeted sanitation, equipment redesign, or physical remediation. In contrast, genetically diverse isolates recovered sporadically are more consistent with repeated upstream introduction from farms, transport, or incoming flocks. Integration with national genomic databases, such as PulseNet and NCBI Pathogen Detection, can also support outbreak detection and traceback. CDC notes that WGS is now the standard PulseNet method for detecting and investigating foodborne outbreaks involving pathogens such as *Salmonella*. Regional and temporal shifts in serovar composition, including the rise of Infantis in US broiler production, are detectable only through this kind of systematic genomic surveillance (Siceloff et al. 2022).

### 12.4 Predictive Modeling and QMRA

Predictive modeling and QMRA can link *Salmonella* concentrations across production, processing, distribution, storage, preparation, and consumption to the probability of human illness (Teunis et al. 2010). These models are valuable because they place preharvest and postharvest interventions on a common public‐health endpoint. For example, a 1‐log reduction in cecal load and a 1‐log reduction at post‐chill are not necessarily equivalent because cross‐contamination, processing interventions, cooking, and consumer handling modify the final risk. Oscar (2021) demonstrated using this framework that prevalence is a poor predictor of risk relative to concentration, providing the quantitative justification for the regulatory shift toward enumeration‐based standards. Risk assessment models may also support future serotype‐focused control by incorporating differences in virulence, exposure, and public‐health relevance among *Salmonella* serotypes.

### 12.5 Decision Support and Implementation

The practical value of analytical tools depends on their translation into operational decisions. Statistical process control applied to quantitative microbial data can help detect process drift before loss of process control or regulatory noncompliance occurs. However, a major implementation challenge in vertically integrated poultry systems is data integration. Breeder, hatchery, feed mill, farm, transport, processing, and product‐lot data are often stored in separate systems and may lack an interoperable flock‐to‐product identifier. Establishing a persistent flock‐level identification system from breeder source to final product lot would improve traceback, source attribution, intervention evaluation, and identification of high‐risk flocks. Such integration would allow producers to prioritize control measures based on biological risk, regulatory relevance, and economic feasibility.

## 13 Conclusion

The One Health concept provides an appropriate framework for *Salmonella* control in poultry production because human health, animal health, food safety, and the production environment are closely connected. *Salmonella* contamination can arise at multiple points in vertically integrated broiler systems, including breeder flocks, hatcheries, feed, water, litter, grow‐out farms, transport, processing, and final product handling. Therefore, effective control cannot rely on a single intervention but requires an integrated farm‐to‐fork strategy targeting both preharvest and postharvest stages.

Preharvest interventions are important because they reduce intestinal colonization, fecal shedding, and the *Salmonella* load entering the processing plant. Breeder‐flock control, hatchery sanitation, biosecurity, litter and water management, vaccination, and targeted dietary interventions can contribute to lowering flock‐level contamination. However, these approaches must be combined with validated processing controls, including hygienic evisceration, chilling, antimicrobial application, post‐chill interventions, and finished‐product verification, to reduce cross‐contamination and consumer exposure.

Although the USDA‐FSIS risk‐based raw poultry *Salmonella* framework was withdrawn in April 2025, its emphasis on microbial concentration, process control, and serotype‐specific public health risk remains scientifically relevant for future control programs. These concepts highlight the need to move beyond simple prevalence testing toward quantitative bio‐mapping, serotype monitoring, whole‐genome sequencing, predictive modeling, and quantitative microbial risk assessment. Such approaches can help identify high‐risk flocks, persistent contamination sources, ineffective control points, and interventions with the greatest public health relevance.

A key conclusion from this review is that reported intervention efficacy must be interpreted cautiously. Large reductions are often observed in controlled challenge studies, but these effects may not translate directly to commercial production. Future research should prioritize field validation, standardized outcome reporting, economic feasibility, and comparisons based on biologically meaningful endpoints such as cecal load, fecal shedding, carcass contamination, product prevalence, and serotype distribution.

Overall, sustainable *Salmonella* control in broiler production will require coordinated application of evidence‐based interventions, routine verification, and improved data integration across the production chain. Establishing stronger flock‐to‐product traceability would improve source attribution, intervention evaluation, and risk‐based decision‐making. This integrated approach is essential for reducing poultry‐associated salmonellosis and improving the safety of poultry products.

## Author Contributions

**Deepak Subedi**: conceptualization, data curation, validation, writing – original draft, writing – review and editing, visualization, software. **Bharath Mallavarapu**: data curation, visualization, writing – original draft, writing – review and editing, software. **Pranita Patil**: writing – original draft, writing – review and editing, data curation. **Abhisek Niraula**: writing – review and editing, writing – original draft, data curation. **Milan Kandel**: data curation, writing – review and editing, writing – original draft. **Sanjay Kumar**: writing – review and editing, writing – original draft, supervision. **Amit Kumar Singh**: supervision, writing – original draft, writing – review and editing.

## Ethics Statement

The authors have nothing to report.

## Conflicts of Interest

The authors declare no conflicts of interest.

## Data Availability

Data sharing is not applicable to this article as no datasets were generated or analyzed during the current study.
